# The structural grammar of integration and competition in the human connectome

**DOI:** 10.3389/fncom.2026.1810942

**Published:** 2026-06-24

**Authors:** Sam Frank Kelemen, Joaquín Goñi, Sérgio Pequito, Arian Ashourvan

**Affiliations:** 1 Department of Psychology, University of Kansas, Lawrence, KS, United States; 2 Edwardson School of Industrial Engineering and Weldon School of Biomedical Engineering, Purdue University, West Lafayette, IN, United States; 3 Institute for Systems and Robotics, Instituto Superior Técnico, Universidade de Lisboa, Lisbon, Portugal

**Keywords:** generative linear model, integrator and mediator hubs, network motifs, structure-function coupling, virtual lesions

## Abstract

**Introduction:**

Brain function emerges from coordinated activity across anatomically connected regions, where structural connectivity (SC)—the network of white matter pathways-provides the physical substrate for functional connectivity (FC), defined as the correlated activity between brain areas. While structural and functional networks exhibit substantial overlap, their relationship involves complex, indirect mechanisms, including the dynamic interplay of direct and indirect pathways. To systematically untangle how structural architecture shapes functional patterns, this work aims to establish a set of *rules* that decode how direct and indirect structural connections and motifs give rise to FC between brain regions.

**Methods:**

Specifically, using a generative linear model, we derive explicit rules that predict an individual's resting-state fMRI FC from diffusion-weighted imaging-derived SC, validated against topological null models.

**Results:**

Examining the rules reveals distinct classes of brain regions, with *integrator* hubs acting as structural linchpins promoting synchronization and *mediator* hubs serving as structural fulcrums orchestrating competing dynamics. Virtual lesion experiments further demonstrate how different cortical and subcortical systems distinctively contribute to global FC.

**Discussion:**

Together, by uncovering how structural architecture governs functional interactions, this framework enables us to predict how alterations in SC, resulting from disease or surgery, propagate through functional networks and contribute to cognitive and behavioral impairments.

## 1 Introduction

Understanding how brain function emerges from anatomical structure represents one of the central challenges in neuroscience. While the neuron doctrine established the foundational principle that neural activity propagates through synaptic connections (Shepherd, 2015), translating this insight to explain large-scale brain dynamics across millions of neurons and complex network architectures remains a formidable challenge. However, this foundational principle scales to extraordinary complexity when considering the stochastic dynamics of neuronal noise, the intricate integration across thousands of dendritic inputs (London and Häusser, 2005), and the hierarchical organization of connectivity patterns that emerge across millions of interconnected neurons spanning multiple spatial and temporal scales.

Given this multi-scale complexity, no unified model currently bridges the dynamics of individual neurons to whole-brain network interactions. Consequently, neuroscience has adopted a levels-based approach, with macroscopic models treating brain regions as abstract nodes governed by phenomenological or biophysically-informed dynamics (Deco et al., 2008). Within this framework, efforts to link structural connectivity (SC) to functional connectivity (FC) have crystallized into three distinct modeling paradigms (Suárez et al., 2020): (i) *statistical approaches* utilize pattern recognition and correlation maximization to identify SC-FC correspondences (Smith et al., 2013; Mišić et al., 2016; Messé et al., 2014), but remain primarily descriptive in revealing areas of convergence and divergence between modalities; (ii) *mechanistic models*, including dynamic systems (Honey et al., 2007; Tanner et al., 2022) and biophysical frameworks (Breakspear, 2017; Sanz-Leon et al., 2015; Deco et al., 2009), simulate the propagation of activity through structural networks to explain the dynamics of the emergent brain, though their interpretations depend heavily on the underlying assumptions about the dynamics of the neural population and the linearity between regions; and (iii) *communication models* bridge these approaches by leveraging network science and graph theory to understand how topology shapes information flow (Mišić et al., 2014, 2015; Graham and Rockmore, 2011; Crofts and Higham, 2009; Becker et al., 2018), operationalizing FC through concepts like shortest-path routing (Goñi et al., 2014), path ensembles (Crofts and Higham, 2009; Avena-Koenigsberger et al., 2018), and transmission-diffusion or coactivation mechanisms (Abdelnour et al., 2014; Mišić et al., 2015).

The evolution of these paradigms reflects a broader progression from descriptive to mechanistic accounts of SC-FC coupling. Early statistical approaches (Messé et al., 2014; Mišić et al., 2016) and subsequent machine learning methods (Sarwar et al., 2021; Zhang et al., 2022; Wei et al., 2024, 2025) established the structural connectome as a critical scaffold for FC, revealing systematic co-variation between structural and functional network architectures. However, these approaches offered limited causal insight into the coupling mechanisms, motivating dynamical and biophysical models (Honey et al., 2007; Breakspear, 2017; Deco et al., 2009) that explicitly simulate signal propagation through structural networks, at the cost of interpretability and computational tractability. Communication models (Goñi et al., 2014; Avena-Koenigsberger et al., 2018; Betzel et al., 2022) occupy an intermediate position, offering principled but simplified accounts of information routing, while typically assuming uniform governing principles across regions and individuals. Each paradigm thus illuminates a different aspect of SC-FC relationships while leaving a key question unresolved: how heterogeneous, region-specific structural rules give rise to the observed diversity of FC patterns across individuals.

In this work, we present a novel approach that combines elements of statistical and communication models. Inspired by the seminal work of Barabási and Barabási (2020) and Kovács et al. (2020), who proposed a generative framework to predict SC in *C. elegans* worms based on their genetic profile, we leverage a similar framework to predict FC in the human brain. Their model utilizes a set of empirically derived *rules* that act linearly on the genetic fingerprint to predict pairwise structural connections between regions. We adapt this framework to predict resting-state fMRI FC between brain regions based on their structural fingerprint measured by diffusion-weighted MRI (DWI). By doing so, we aim to demonstrate how both direct and higher-order connection patterns and motifs between neurons contribute to the observed patterns of functional connectivity. Subsequently, we will utilize the derived rules to explore the influence of various cortical and subcortical regions on large-scale correlation and anti-correlation patterns across the cortex.

## 2 Results

**Figure 1:**
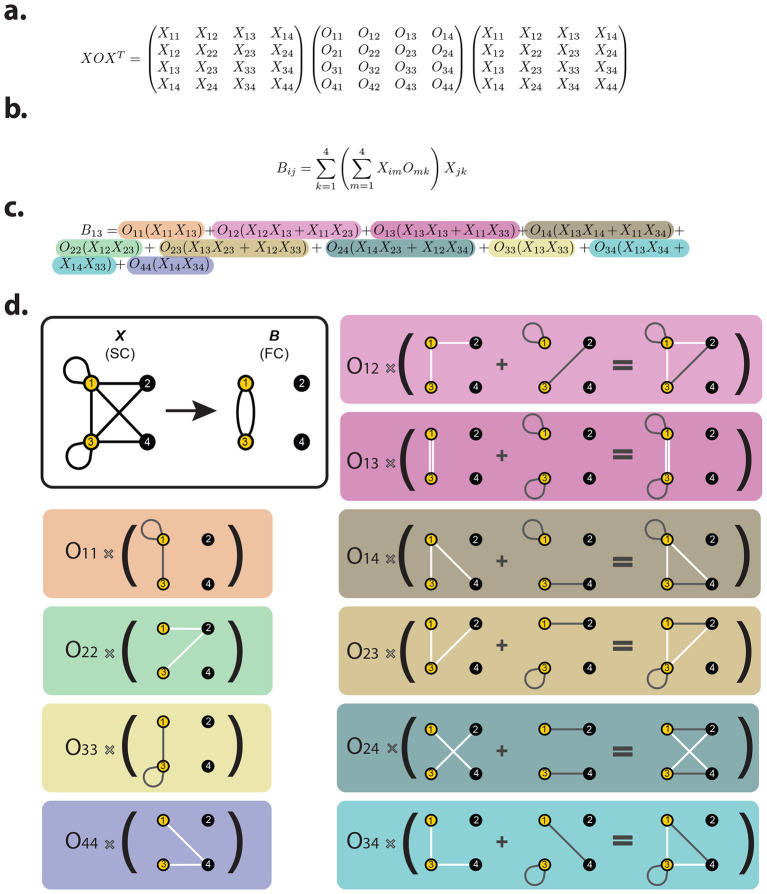
Network motifs and connectivity patterns captured by structural generative rules. **(a)** The equation for generating the 4 × 4 symmetric FC matrix (*B*) from the SC matrix (*X*) using the generative rule matrix (*O*). Since *O* is symmetric (see Section 4 for details), the element-wise equation for *B* can be represented as depicted in **(b)**. **(c)** Expanded equation illustrating the generation of FC between nodes 1 and 3 using the corresponding elements of *X* and *O*. The color-coded sections highlight various connection patterns and motifs, as depicted in **(d)**, contributing to the FC between nodes 1 and 3.

Let FC be represented by the symmetric matrix *B*∈ℝ^*N*×*N*^ and SC by the symmetric matrix *X*∈ℝ^*N*×*N*^, where *N* is the number of brain regions. We propose a linear generative model given by the equation *B* = *XOX*^*T*^, where *O*∈ℝ^*N*×*N*^ is the symmetric *rule* matrix that captures how SC patterns with other regions influence the FC weight between two regions. In Figure 1, we illustrate how the elements of the rule matrix encode various direct and monosynaptic structural connection patterns and how their linear combinations contribute to the FC weight between two regions.

### 2.1 Predicting FC from individuals' SC

#### 2.1.1 Subject-level rule estimation and prediction accuracy

**Figure 2:**
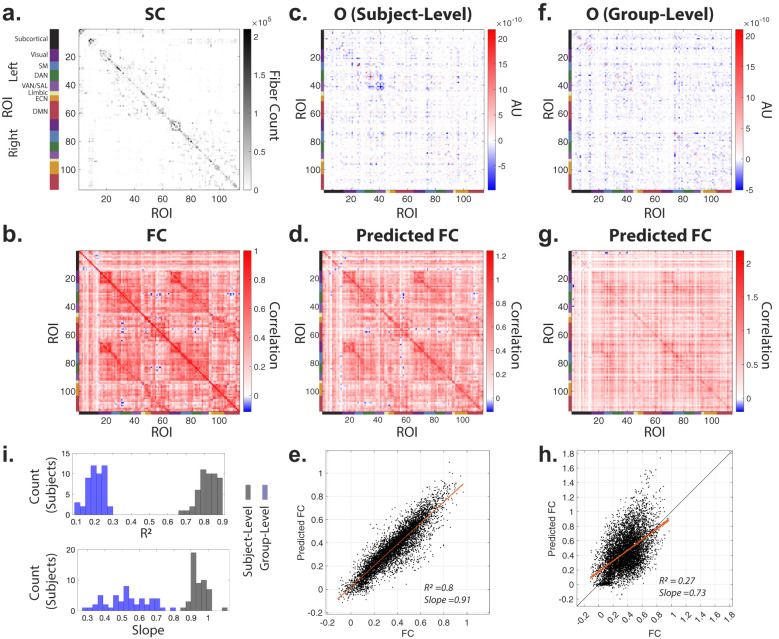
Predicted FC from SC. **(a)** Sample subject's SC matrix and **(b)** corresponding FC matrix. **(c)** The estimated subject-level rule matrix, highlighting significant values compared to a randomized null model with preserved degree distribution (*p* < 0.05, FDR corrected for multiple comparisons), and **(d)** the predicted FC matrix using the rules from **(c)**. **(e)** Scatter plot comparing the sample subject's actual FC values with the predicted FC values, and the red line indicates the linear fit. **(f)** The estimated group-level rule matrix, highlighting significant values compared to a randomized null model, and **(g)** the predicted FC matrix for the same sample subject using the group-level rules from **(f)**. Here, the predicted FC values equal to or higher than one are represented with the same color to aid visualization. **(h)** Scatter plot comparing the sample subject's actual FC values with the predicted FC values using the group-level rule matrix from **(f)**. The red line shows the linear fit, and the dashed line the 95% Confidence interval. **(i)** Distributions of the *R*^2^ values (top) and slopes of the linear fit between the actual and predicted FC values using the subject- (black) and group-level (blue) models.

In Figure 2, we present the estimated rule matrix and the predicted FC values for a sample subject using LASSO regression—see Section 4 for details. The model demonstrates high accuracy in predicting FC values, as evidenced by the close linear fit between the original and predicted values shown in Figure 2e. Across all subjects, the model achieves an average slope of 0.94 ± 0.04 and an average *R*^2^ of 0.81 ± 0.05.

To examine whether the relationship between SC and FC is better characterized by linear or logarithmic scaling, we compared model predictions using fiber count SC against those using log_10_(SC+1). Predictions using log-transformed SC resulted in a significant reduction in both the mean goodness-of-fit (*R*^2^: *t*-test, *p* = 4.8 × 10^−18^) and the slope (*t*-test, *p* = 2 × 10^−38^) of the linear regression between actual and predicted FC values across all subjects (see Supplementary Figure 1). These results support the use of fiber counts rather than log-transformed values as input to the generative model.

#### 2.1.2 Group-level rule estimation reveals universal principles

To uncover universal rules for predicting FC from subjects' SC patterns and the extent of intersubject variability, we fit a single rule matrix *O* to all subjects' SC and FC matrices at the group level. We leveraged the Kronecker product and inverted the resulting group-level SC matrix—see Section 4 for details.

In Figure 2g, we show the predicted FC for a sample subject using the group-level rule matrix presented in Figure 2f. The linear fit between the empirical and predicted FC values in Figure 2h indicates that group-level rules can indeed generate FC, with an average slope of 0.53 ± 0.12 and *R*^2^ of 0.2 ± 0.04 across all subjects. However, compared to the subject-level rules, the group approach results in a significant (*t*−test, *p* = 2 × 10^−48^) reduction in the goodness-of-fit of the linear regression fit between empirical and predicted FC.

#### 2.1.3 Rule matrix sparsity and robustness analysis

**Figure 3:**
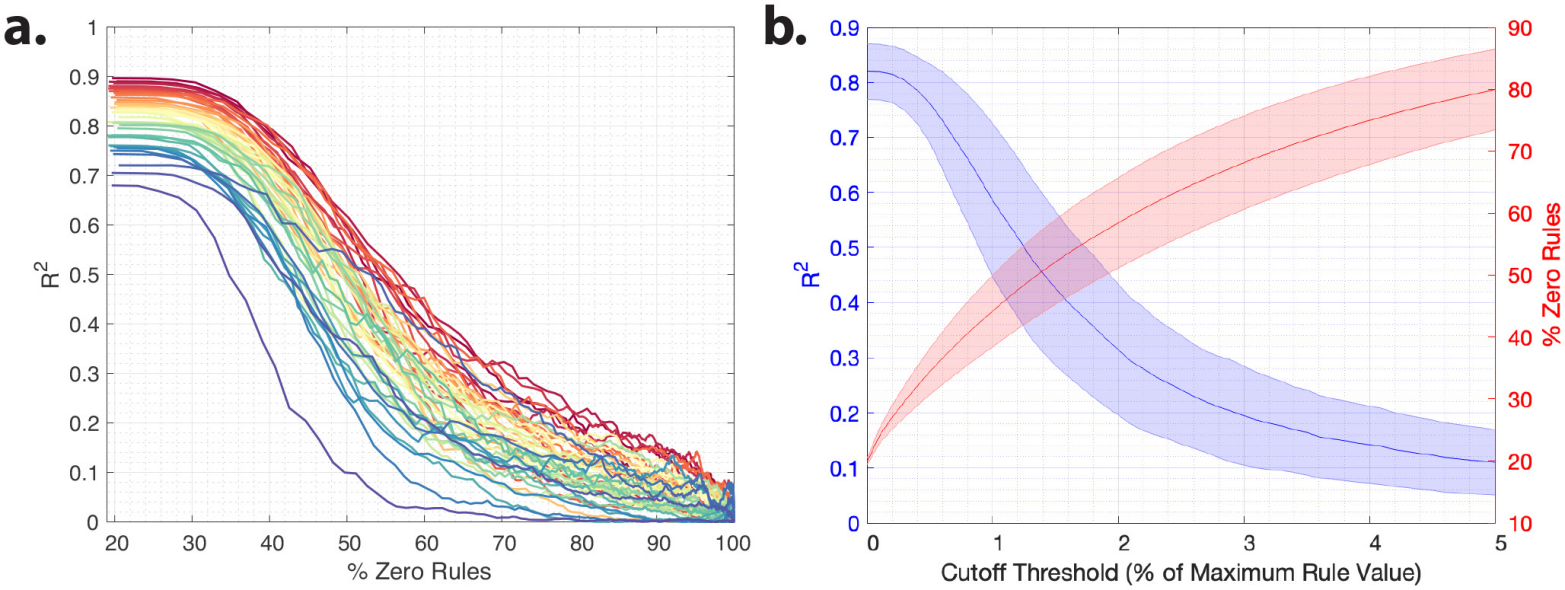
Impact of thresholding the rule matrix on prediction accuracy. **(a)** The model's accuracy is measured by the goodness-of-fit of a linear regression fit, indicated by *R*^2^, comparing the actual vs. predicted FC values. Each color-coded line represents the *R*^2^ value associated with each subject as we increase the threshold, replacing the elements of the rule matrix *O* below the cutoff with zero. The x-axis represents the total percentage of zero elements in the rule matrix as we increase the threshold. **(b)** The shaded blue line indicates the averaged *R*^2^ values across subjects, while the thickness of the shaded area reflects the standard deviation. The x-axis displays the cutoff threshold values as percentages, relative to the maximum rule element value. The red line represents the average proportion of zero elements relative to the total number of elements in percentage at each cutoff threshold.

The rule matrix exhibits a heavy-tailed weight distribution, with a high density of zero values—see Supplementary Figure 2. To assess the sensitivity of the model predictions to these smaller-weighted rules, we incrementally replaced the lowest-valued rules with zero and evaluated the resulting prediction accuracy (Figure 3). The prediction accuracy remained relatively stable until the group-average proportion of zero elements reached approximately 30% of the total rules (Figure 3b), after which it sharply declined.

#### 2.1.4 Subject-specific parameters do not generalize across individuals

Human brain networks have universal properties (small-world, modular, heavy-tailed degree distributions) despite individual structural differences. To investigate how a subject's unique structural network influences the prediction of functional connections, we compared predictions made using their own SC with those made using another subject's SC, both using the same model parameters.

Our results indicate that the subject-specific model parameters are highly individualized and do not generalize well to other subjects' SC (Supplementary Figure 3). We calculated the predictive accuracy of the model by performing a linear regression of the predicted vs. observed FC values. The significant drop (*t*−test, *p* = 8.1 × 10^−61^) in model accuracy (i.e., *R*^2^) when predicting FC using another subject's SC, compared to using the subject's own, underscores this finding (Supplementary Figure 3a).

For each subject, we also evaluated the predictive accuracy of the model when we swapped the subject's SC with that of edge-randomized SC null models, preserving the degree and strength distributions of each brain region. Similarly, the predictions are highly inaccurate when we replace the SC with SC nulls (*t*−test, *p* = 8.1 × 10^−^61).

#### 2.1.5 Topological features preserve predictive performance

We repeated the previous analyses, but with a change in how the rule matrices (*O*) were estimated. Instead of using each subject's own SC and FC matrices, we estimated *O* using another subject's SC matrices. Our results indicate that fitting the model parameters leads to highly accurate predictions, even when using data from another subject's SC or null SCs (Supplementary Figures 3c, d). Statistical testing confirmed that the results are indistinguishable at the group level. Supplementary analysis at the individual level revealed an inconsistent pattern of performance that was either comparable to, lower than, or higher than the null expectations across subjects, as shown in Supplementary Figure 4. It reveals that a subset of subjects does not exhibit higher prediction accuracy than the topological null model when *O* is fitted to another subject's SC. This inconsistency reflects two compounding factors. First, genuine inter-individual variability in which unique structural fingerprints matter more for some subjects, so that topological features alone are insufficient to capture their FC. Second, the possibility that subject-level overfitting contributes to the performance gap, particularly for subjects where *O* may have fit session-specific FC noise. Combined with the subject- vs. group-level accuracy gap (*R*^2^ = 0.81 vs. 0.20), these results reinforce the importance of individualized modeling and the need for prospective validation (see Section 3.4). At the group level, the SC null model provides the primary test of structural specificity. Fitted-*O* accuracy with null SC is far below that with true SC, confirming that the group-average rule captures genuine structure-function coupling rather than noise.

#### 2.1.6 Statistical significance of rule elements

We assessed the model's accuracy after retaining only the rules whose values exceeded those estimated from the edge-randomized SC null model. To identify significant rule elements at the subject-level, we used nonparametric permutation testing (*p* < 0.05, FDR-corrected for multiple comparisons) using *n* = 100 randomized SC null models. On average, more than 4% of rule elements did not pass the significance level (Supplementary Figure 5a). Importantly, by removing the non-significant elements results in a small, yet significant (*t*−test, *p* = 1.01 × 10^−14^), reduction in the model prediction accuracy (Supplementary Figure 5b). Together, these results indicate that preserved topological features, such as the degree sequence, play a crucial role in shaping functional connectivity predictions.

#### 2.1.7 Direct vs. indirect structural features in FC prediction

We compared the model's predicted FC using SC with predictions based on search information (Goñi et al., 2014), a metric that captures the navigability of shortest paths across the network – see Section 4 for details. This comparison allows us to assess whether functional interactions are better explained by direct anatomical links or by the ease of communication through the broader network topology, which is an important distinction for identifying the mechanisms underlying functional coupling.

Our results show that models driven by each subject's own SC achieve markedly higher predictive accuracy, as reflected in significantly larger *R*^2^ values (Supplementary Figure 6). This implies that the direct, monosynaptic connections represented in the SC already account for the bulk of the FC variance. While search information can capture part of the residual variance, replacing SC with the search information matrix as input in our generative model diminishes its ability to represent the effect of the immediate structural links accurately.

### 2.2 Organizing the brain by structure-function coupling roles

#### 2.2.1 Disentangling direct and structural motif contributions to FC

The rule matrix *O* quantifies how direct SC and first-order indirect SC pathways collectively shape FC. The prevalence of high diagonal values across all regions highlights the importance of direct SC and first-order indirect pathways in shaping FC. These findings remain robust even after removing non-significant rules at the subject level, with only an approximately 2% difference in the identification of mean significant rules across all subjects (Supplementary Figure 5). However, the presence of off-diagonal weights implies that structural motifs (see Figure 1d) also contribute to FC.

#### 2.2.2 Rule matrix organization reveals structural linchpins and fulcrums shaping FC

**Figure 4:**
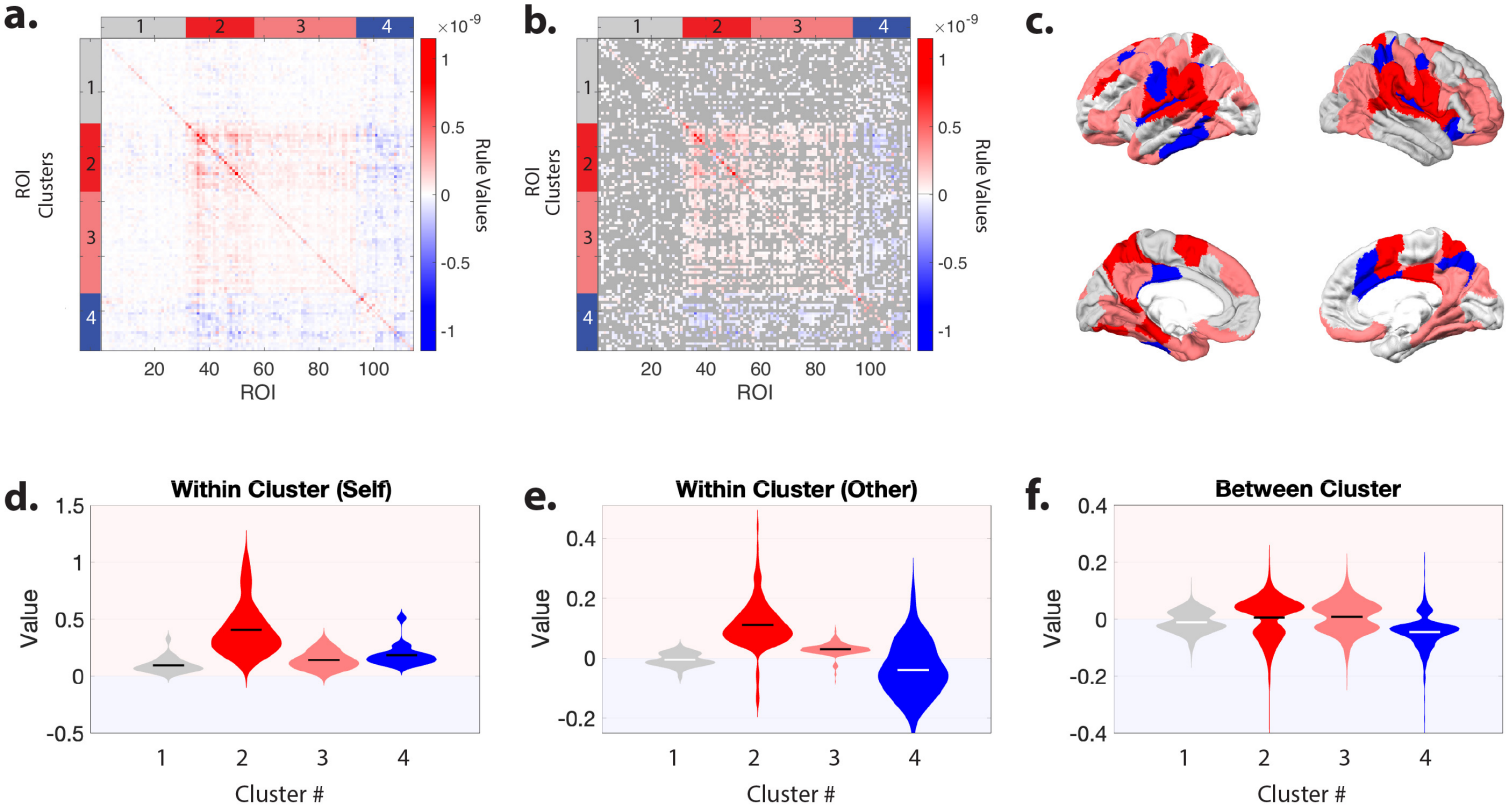
Structural linchpins and fulcrums of functional dynamics. **(a)** Subject-level rule matrix *O* averaged across all subjects, with brain regions sorted by their cluster assignments identified using the WSBM method (*k* = 4). **(b)** Same matrix in **(a)**, except the rule elements with means that show no significant (*t*−test, *p* < 0.05, FDR-corrected for multiple comparisons across all rules) difference from zero across all subjects are color-coded in gray. **(c)** Brain overlay of the identified clusters. Distributions of the significant within-cluster self-connections (diagonal elements) **(d)**, within-cluster other connections (off-diagonal elements) **(e)**, and between-cluster connections **(f)** from the matrix in **(b)**, color-coded according to their community assignments. Black (white) bars show the means of distributions with positive (negative) signs.

To elucidate the structural roles of brain regions in driving these dynamics, we applied weighted stochastic block modeling (WSBM)—see Section 4 for details – to partition the *O* averaged across all subjects into four clusters based on similarities in their within- and between-cluster connection profiles (Figure 4).

Our approach revealed distinct organizational principles: Cluster 1 exhibited weak off-diagonal interactions. Cluster 2 and, to a lesser extent, Cluster 3 displayed positive intra- and inter-cluster weights, indicative that contributions from indirect SC motifs enhance FC. In contrast, Cluster 4 was uniquely characterized by globally negative off-diagonal weights, suggesting structural motifs involving this cluster's brain regions suppress FC.

**Figure 5:**
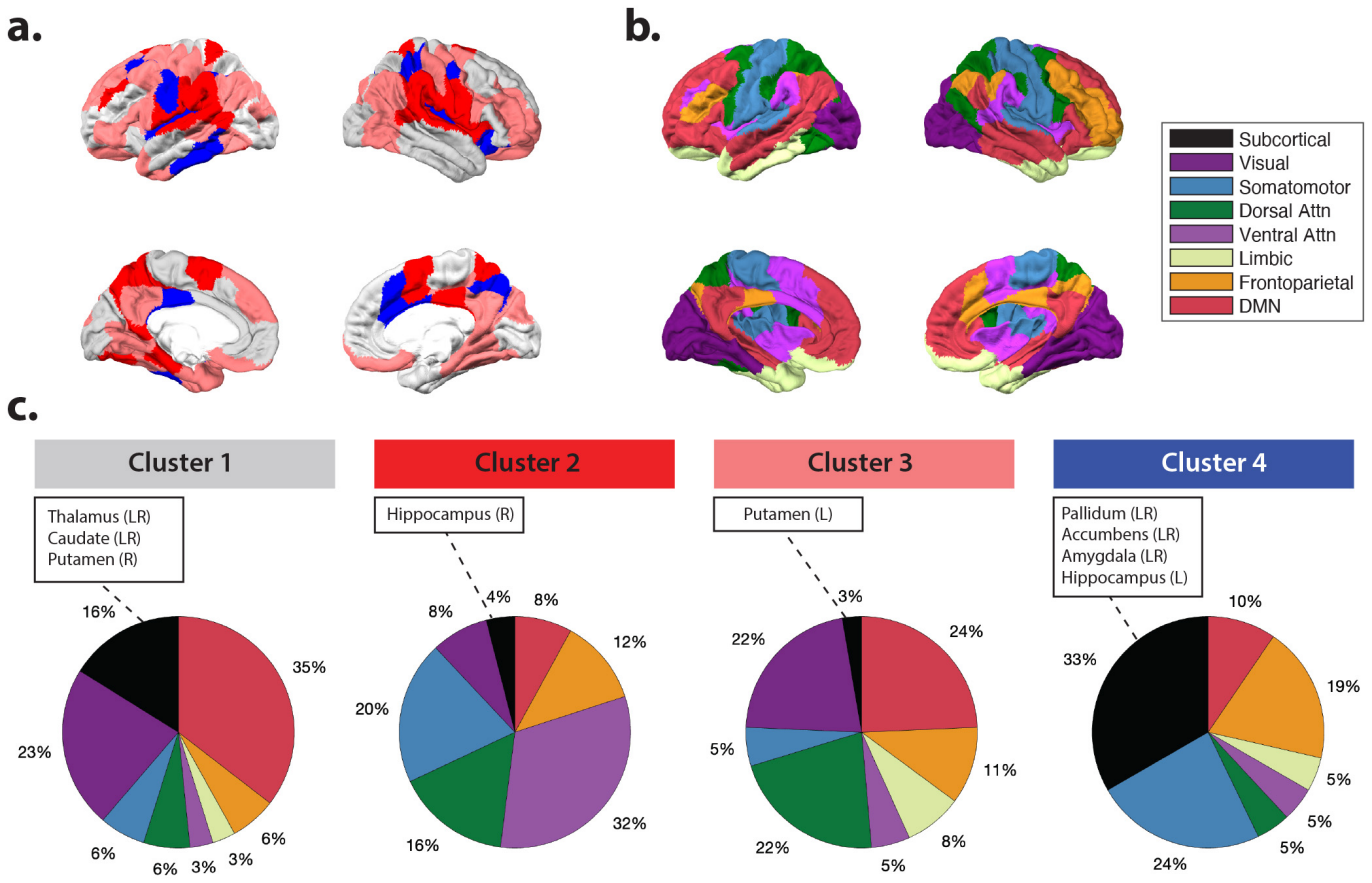
Rule matrix clusters overlap with resting-state networks. **(a)** Brain overlay of clusters identified from the mean rule matrix *O* in Figure 4, juxtaposed with the seven resting-state brain networks. **(b)** Resting-state networks as identified by (Thomas Yeo et al. 2011). **(c)** Percentage contribution of resting-state ROIs to the identified clusters.

Additionally, we mapped each cluster's ROIs to seven canonical resting-state networks and subcortical regions (Figure 5). This analysis revealed heterogeneous contributions across clusters. For example, nearly half of the regions of interest in Cluster 2 were from attention networks, while Cluster 4 primarily comprised subcortical, somatomotor, and frontoparietal ROIs.

Together, these results show that WSBM-based decomposition of the rule matrix *O* delineates distinct structural classes, such as *mediator* (Cluster 4) and *integrator hubs* (Clusters 2-3), whose motif-mediated structural interactions shape large-scale functional dynamics. Integrator hubs serve as *structural linchpins*, promoting synchronization among the regions they connect, while mediator hubs function as *structural fulcrums*, facilitating competitive dynamics between their connected regions. Together, these findings provide a taxonomy of brain regions based on how structural specialization shapes emergent functional connectivity in the brain.

#### 2.2.3 Inter-individual variability and reliability of the group-average rules

To assess whether the structural grammar reflects conserved biological organization rather than subject-specific noise fitting, we performed WSBM on each subject's individual *O* matrix (*k* = 4, 10 trials per subject, best log-evidence solution retained; community labels aligned to the group solution via the Hungarian algorithm). Pairwise NMI across subjects (0.128 ± 0.037) significantly exceeded a null distribution of randomly permuted labels (0.030 ± 0.016; one-tailed *t*-test, *p* < 10^−30^), confirming that subjects share substantially more partition structure than expected by chance (Supplementary Figures 7a, b). Critically, the sign of each cluster-to-cluster interaction block in *O* was conserved across 68–94% of subjects (8 of 10 block pairs FDR-significant; *t*-test; Supplementary Figure 7d). The negative C1–C4 block (92% of subjects, *t* = −6.87) and positive C2–C3 block (82%, *t* = 6.09) directly correspond to the mediator and integrator roles identified in the group analysis and are highly reproducible (Supplementary Figure 7e). Node-level consistency differed significantly across clusters (Kruskal-Wallis, *p* < 0.0001; Supplementary Figure 7c), confirming structured rather than random variability. These results support the interpretation that the block-sign grammar is a biologically reproducible property of the human connectome, while the subject-specificity of node assignments reflects genuine individual differences in structural architecture.

### 2.3 Examining the impact of regional structural profiles on whole-brain FC

**Figure 6:**
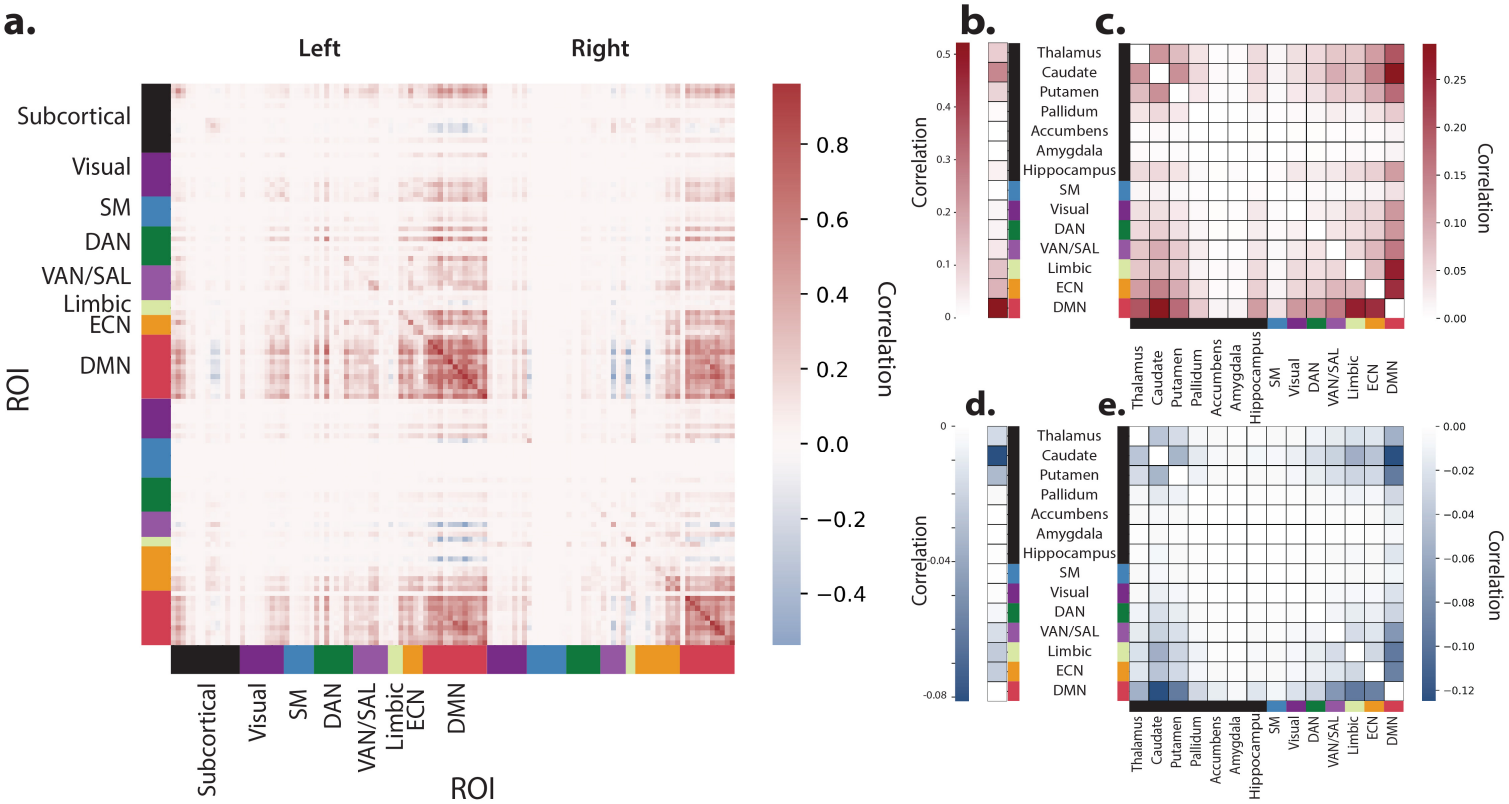
Default mode network's influence on global FC patterns. **(a)** Predicted FC matrices after selectively removing the influence of all regions except for the DMN regions of interest. **(b)** Average predicted positive FC values within each ROI for different systems, and **(c)** between different ROIs for different systems. **(d)** Average predicted negative FC values within each ROI for different systems, and **(e)** between different ROIs for different systems.

Our generative framework provides a principled way to tease apart how individual regions or networks shape whole-brain FC. We accomplish this by retaining only those SC edges that originate from or terminate in a chosen target region or network. Leveraging the group-level rule matrix *O*, we demonstrate how the framework quantifies each region's specific contribution to global FC. As a proof of concept, we simulate the effect of selectively preserving only the default mode network (DMN) structural connections (Figure 6), demonstrating that our model yields viable and informative predictions. Figure 6 illustrates the group-average contribution of the default mode network (DMN) to FC. The DMN's SC not only directly shapes intra-DMN FC and the coupling between DMN nodes and extra-DMN regions, but also indirectly modulates FC among pairs of non-DMN regions via their shared connections with DMN ROIs. This demonstration highlights how our framework can be used to systematically assess the impact of specific regions and networks on global FC. More importantly, it underscores the model's utility for studying how localized structural disruptions, such as those resulting from aging, neurodevelopmental disorders, traumatic brain injury, or surgical resection, alter whole-brain functional organization.

### 2.4 Pathological changes in the structure-function coupling rules

Changes in the coupling between SC and FC have been observed across various neurological and psychiatric conditions, including schizophrenia (SZ) (Zhao et al., 2023), Major Depressive Disorder (Liao et al., 2024), and Autism Spectrum Disorder (Qing et al., 2024). In SZ, SC-FC coupling exhibits a complex and regionally specific pattern of alterations, with some brain networks demonstrating increased coupling while others exhibit decreased coupling (Sun et al., 2017). These disruptions are thought to contribute to the aberrant neural dynamics underlying the cognitive and clinical symptoms characteristic of the disorder. Therefore, we hypothesized that our framework can identify the structural determinants of FC alterations by analyzing the estimated rule matrix *O*.

**Figure 7:**
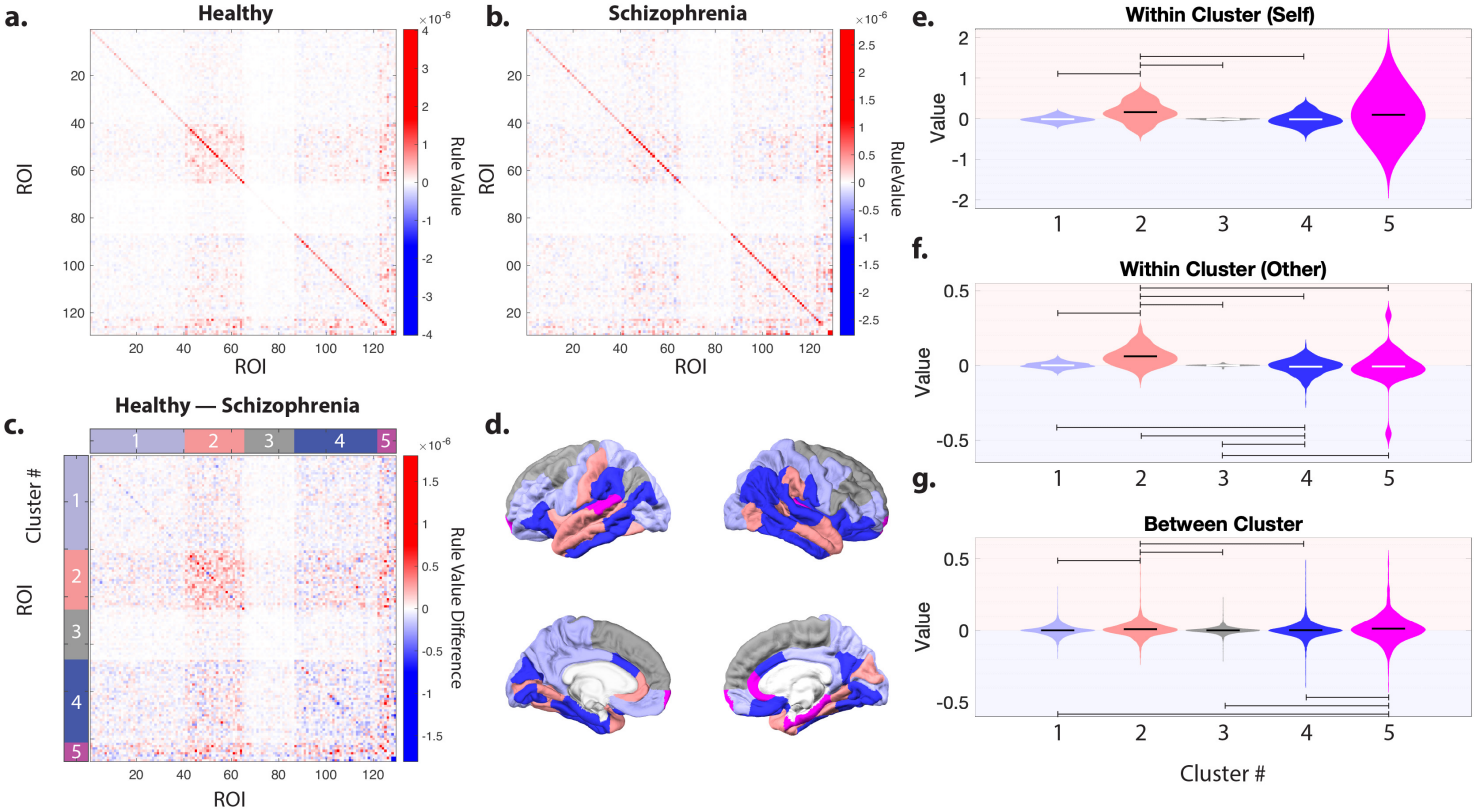
Pathological changes in the SC-FC coupling rules. The group-average rule matrices *O* for healthy controls **(a)** and individuals with SZ **(b)**. **(c)** Difference matrix (healthy minus SZ), with rows and columns ordered by WSBM-derived cluster assignments (*k* = 5). **(d)** Brain surface overlay depicting the spatial distribution of the five WSBM clusters shown in **(c)**. Distributions of rule values extracted from the difference matrix in **(c)**: **(e)** within-cluster self-connections (diagonal elements), **(f)** within-cluster off-diagonal connections, and **(g)** between-cluster connections. Violin plots are color-coded by the WSBM community; black bars indicate the mean of positive-valued distributions, and white bars indicate the mean of negative-valued distributions.

To test this hypothesis, we utilized a publicly available structural and functional connectome dataset comprising SZ patients and healthy control participants—for details on the dataset see Section 4. We then assessed differences in the estimated rule matrices across SZ and control groups. Figure 7 presents the average rule matrix *O* for both the healthy control and SZ groups. Organizing SZ vs. healthy control differences in the group-average rule matrices with a WSBM method highlights pronounced spatial heterogeneity in SC-FC coupling. Choosing *k* = 5 blocks maximizes interpretability of between-group contrasts (Figure 7c). The strongest alterations cluster within the temporal lobe. Cluster 2, which carries the largest positive rule weights in controls, is significantly attenuated in SZ (Wilcoxon rank-sum, *p* < 0.05 vs. nearly every other cluster). By contrast, Cluster 4 shows the opposite trend, with rule weights shifting toward more negative values in SZ.

Finally, a compact bilateral set of ROIs, including the auditory cortices, frontal pole, right anterior cingulate cortex, and medial temporal regions, exhibits the greatest absolute deviations in rule weights across the brain, underscoring focal SC disruptions that may drive widespread functional alterations in SZ. Together, these findings demonstrate how our framework can uncover structured, network-level alterations in SC-FC coupling, providing a powerful lens to study the systems-level impact of brain disorders such as schizophrenia.

## 3 Discussion

The intricate relationship between structural and functional connectivity in the human brain remains a central focus in network neuroscience and computational neuroimaging. While previous approaches have either relied on purely statistical correlations or complex biophysical simulations with limited interpretability, our generative linear framework offers a systematic approach to elucidate the topological and dynamical principles governing this relationship.

By integrating DWI-derived SC and resting-state fMRI FC, our study contributes to the ongoing efforts to map the structural underpinnings of functional communication in the human brain. Specifically, our results provide mechanistic insights into structural determinants of FC through both direct and monosynaptic pathways and reveal organizational roles wherein specific regions act as structural *linchpins* and *fulcrums*, supporting global functional dynamics.

### 3.1 Mechanistic insights from structure-function coupling rules

#### 3.1.1 The role of structural motifs in FC

The rule matrix *O* in our framework provides a data-driven characterization of how direct and first-order indirect structural connections, along with mono-synaptic motifs, shape FC across the brain. The prominence of off-diagonal elements in the rule matrix suggests that more complex interactions, such as mono-synaptic motifs involving multiple regions, are essential to explain the observed FC beyond what can be accounted for by direct and first-order indirect SC alone.

These interactions establish the structural scaffolding for FC to emerge through long-distance synchronization among multiple regions, a mechanism theorized in communication models emphasizing path ensembles and polysynaptic signaling (Avena-Koenigsberger et al., 2017, 2018; Goñi et al., 2014). For instance, the dorsal and ventral attention networks, along with somatomotor regions, emerged as dominant contributors to functional dynamics supported by these structural motifs, aligning with attention networks' role in coordinating spatially distributed information processing (Corbetta and Shulman, 2002).

#### 3.1.2 Cortical and subcortical regulation of functional anti-correlations

We provided evidence that anti-correlations or reduced positive FC were mediated indirectly through structural motifs mainly involving several cortical regions within networks, such as somatomotor and frontoparietal networks, and subcortical regions, including bilateral nucleus accumbens (NAc), globus pallidus (GP), and amygdala.

Subcortical regions such as NAc and GP contribute to large-scale functional connectivity through GABAergic polysynaptic loops involving the thalamus and prefrontal cortex. These regions act as anatomical and functional bottlenecks, integrating diverse inputs and modulating network activity via inhibitory signaling, which may suppress or prioritize information flow across cortical systems (Bell and Shine, 2016; Zhao et al., 2024).

Experimental and clinical studies, particularly those using deep brain stimulation in neuropsychiatric disorders, suggest that perturbations to the NAc and globus pallidus internus (GPi) can alter FC profiles across cortico-subcortical networks (Figee et al., 2013). These changes often manifest as amplification or disruption of positive correlations (e.g., hyperconnectivity in cortico-striatal circuits) or as reduced anti-correlations between networks, such as the default-mode and task-positive systems.

For example, experimental modulation of the ventral pallidum has been shown to toggle large-scale dynamics between task-positive and default-mode network states (Klaassen et al., 2021). While direct causal evidence for cortical regions' on global FC modulation remains limited, studies of neuropsychiatric disorders such as depression provide evidence for increased FC to cortical regions, including frontoparietal networks implicated in cognitive control, reflecting impaired top-down regulatory control (Sheline et al., 2010; Zhou et al., 2010).

#### 3.1.3 Emergence of anti-correlations through network dynamics

Despite strong evidence for the role of inhibitory mechanisms, large-scale modeling studies have shown that anti-correlations in FC can emerge even in the absence of explicitly negative structural connections. A variety of modeling frameworks—including neural mass models, mean-field approximations, and dynamic mean-field models—have demonstrated that factors such as time delays, oscillatory phase differences, and global coupling strength can induce anti-correlated activity between regions that are structurally connected through predominantly excitatory pathways (Deco et al., 2009; Cabral et al., 2011; Hellyer et al., 2014).

These phenomena reflect emergent network-level dynamics, whereby ensembles of regions become functionally segregated through collective behavior rather than direct inhibitory wiring. Thus, caution is warranted when interpreting high-magnitude rule weights as simple direct proxies for the density of structural connections, or negative weights as definitive evidence of inhibitory projections. The negative off-diagonal weights in Cluster 4 are best interpreted as net suppressive influences on FC mediated through structural motifs, rather than direct evidence of physiological inhibition. Anti-correlations in BOLD FC can emerge from purely excitatory structural networks through mechanisms including time delays, oscillatory phase differences, and global coupling dynamics (Deco et al., 2009; Cabral et al., 2011; Hellyer et al., 2014). The sign of *O*_*ij*_ therefore reflects the net direction of structural motif contributions to FC, not the sign of the underlying synaptic connectivity. Causal identification of the mechanisms driving suppressive motif interactions would require perturbational or effective connectivity approaches beyond the present scope. Instead, the rules should be inferred as capturing the net effect of multi-scale mechanisms, including microcircuit properties, mesoscale architectural constraints, and large-scale dynamical interactions that jointly shape the observed FC between distant brain regions.

#### 3.1.4 Primacy of direct structural connections over global topological features

Despite an incomplete understanding of all underlying mechanisms, our results provide strong evidence for the importance of direct SC and monosynaptic structural motifs in shaping the observed FC. Specifically, we found that replacing SC with SC-derived matrices, such as search information, which quantifies the ease with which information can traverse the shortest path between two nodes, significantly reduces the model's predictive performance.

This indicates that incorporating shortest-path-based metrics, which integrate global topological information, may obscure or dilute the local, monosynaptic features that are critical for accurate FC prediction. These findings suggest that, unlike unbiased path-based features of SC, our framework's predictive power stems from its ability to estimate the heterogeneous and localized effects of direct SC connections across the brain for each subject in shaping FC. Search information was selected as the comparison metric because it operationalizes the dominant alternative hypothesis—specifically that FC is shaped by global network navigability—in the most principled available form. Among path-based communication metrics, search information is the most directly interpretable as a navigation cost and has well-established precedent for SC-FC prediction (Goñi et al., 2014). The consistent superiority of raw SC over search information supports the primacy of local, monosynaptic communication in this regime. A systematic evaluation of all available communication metrics is beyond the present scope; the key distinction our comparison tests is between models that respect the local, monosynaptic structure of SC vs. those that aggregate global path information. The superiority of raw fiber counts over log-transformed SC has both computational and biological implications. Computationally, *B* = *XOX*^*T*^ is bilinear in *X*, meaning each FC element is a weighted sum of products *X*_*im*_·*X*_*jk*_. A rule *O*_*mk*_ therefore contributes to FC between regions *i* and *j* in proportion to the product of their tract strengths to *m* and *k*. Logarithmic compression collapses this dynamic range, making a 1000-fiber tract appear nearly as strong as a 10-fiber tract [log(1001)≈3.0 vs. log(11)≈2.4], so *O* can no longer resolve which structural motifs actually drive strong vs. weak FC. Biologically, raw fiber counts scale with axonal cross-sectional volume and, by extension, with synaptic drive (Honey et al., 2007). This is consistent with linear noise models in which spontaneous BOLD covariance is proportional to anatomical coupling strength (Deco et al., 2013).

Taken together, our findings underscore that preserving the fine-grained, subject-specific architecture of direct anatomical links yields a markedly more faithful and mechanistically interpretable account of whole-brain functional dynamics than substituting those details with abstract path-based statistics.

### 3.2 Generative models of SC-FC couplings

#### 3.2.1 Historical evolution of structure-function modeling approaches

The three modeling paradigms reviewed in the Introduction (statistical, dynamical/biophysical, and communication) each illuminate distinct aspects of SC-FC coupling while leaving a shared gap: none fully accounts for the heterogeneity of coupling rules across brain regions and individuals (Messé et al., 2015; Li and Yap, 2022). Stochastic and maximum entropy approaches (Watanabe et al., 2013; Ashourvan et al., 2021) and dynamical systems models (Demirtaş et al., 2019; Tanner et al., 2024; Breakspear, 2017; Honey et al., 2007; Herzog et al., 2024) provide mechanistic depth but limited interpretability of region-specific rules. Communication models offer interpretable motifs (Crofts and Higham, 2009; Goñi et al., 2014; Avena-Koenigsberger et al., 2018; Betzel et al., 2022) but assume homogeneous propagation principles. The present work addresses this gap by deriving explicit, individualized coupling rules that are both mechanistically grounded and directly interpretable at the region and motif level.

#### 3.2.2 Bridging interpretability and accuracy

Our work introduces a generative, individualized framework that achieves high accuracy in predicting FC, while retaining high interpretability. Critically, we demonstrate how structural motifs, such as triangular connections, shape the balance between integration and segregation in global functional dynamics. These findings provide mechanistic insights into how localized anatomical interactions scale to system-wide phenomena.

Furthermore, despite the large number of model parameters, SC null testing revealed that the model's ability to accurately predict an individual's FC is highly specific to their own SC. This finding strengthens our confidence that the results reflect genuine coupling rules rather than overfitting. Together, these findings suggest that a simple monosynaptic communication model, which effectively captures intersubject variability and heterogeneity in coupling rules, can robustly explain the emergence of global FC patterns.

Our findings also show accurate FC predictions even when using a different subject's SC or a randomized SC null model, provided the model parameters are re-estimated. This suggests that aligning an individual's structural and functional connectomes isn't crucial for high predictive performance, as long as the SC's topological features are preserved.

Our findings also underscore the individual variability in SC-FC coupling by demonstrating the superior predictive power of subject-specific rules over group-level rules. This observation reinforces the findings from connectome identifiability (Finn et al., 2015; Amico and Goñi, 2018) and other research demonstrating that personalized connectome architecture accounts for unique functional dynamics (Cooper et al., 2024), suggesting that group-level models may overlook critical idiosyncrasies in brain organization.

### 3.3 Clinical and translational impact

#### 3.3.1 Framework for forecasting functional impact of structural alterations

Understanding how the effect of structural disconnections propagates through functional networks has direct implications for clinical applications (Luppi et al., 2023). Our framework provides a principled method for quantifying the functional impact of focal structural lesions, such as stroke, traumatic brain injury, and neurodegenerative disorders.

Previous studies have underscored the critical role of white matter integrity in maintaining cognitive function, showing that disruptions in the coupling between structural and functional connectivity serve as key indicators of cognitive impairment (Popp et al., 2024; Wang et al., 2018) and pathological conditions (Zhang et al., 2021; Ma et al., 2021).

Our findings in the schizophrenia dataset reveal a clear disruption in the normal coupling patterns between structural and functional connectivity, particularly within temporo-limbic and frontoparietal circuits. These alterations are consistent with prior evidence showing that SC in SZ is characterized by reduced white matter integrity, particularly in associative tracts, such as the Cingulum bundle, Uncinate fasciculus, and Superior longitudinal fasciculus, linking the frontal and temporal lobes (Kubicki et al., 2007; Dabiri et al., 2022). A pivotal finding of our study is that alterations in SC in pathology are not the sole determinants of observed pathological FC. Instead, our results compellingly demonstrate that the SC-FC coupling rules themselves undergo changes in the SZ group.

This rule alteration can be conceptualized by considering that if SC between a pair of regions is diminished or absent in pathology, the algorithm will inherently assign minimal rule weight (especially with regularization) to predict their FC. Conversely, an increase in certain rule weights indicates a magnified contribution from the associated SC, potentially reflecting either an actual increase in SC or, critically, pathological compensatory mechanisms that lead to an observed increase in FC. Although a comprehensive elucidation of the neurobiological meaning of these SZ-related changes in FC is beyond the present scope, our work establishes a new frontier for exploring how SC-FC coupling rules are impacted by aging or various pathologies (Li et al., 2024).

#### 3.3.2 Applications in neurosurgical planning

In addition, our model uniquely extends current understanding by offering predictive insights into the causal links between specific SC disruptions and large-scale FC reorganization. One highly promising application lies in preoperative neurosurgical planning. Virtual lesion experiments have been used to estimate post-surgical functional outcomes in patients with epilepsy (Jirsa et al., 2023), stroke (Idesis et al., 2024), and tumor resection patients (Aerts et al., 2020). Our generative model holds the potential to refine predictions by explicitly accounting for subject-specific SC-FC relationships, leading to enhanced prognostic accuracy.

By systematically comparing SC-FC coupling across a range of neurological and psychiatric disorders, future research could pinpoint disorder-specific alterations in structure-function rules. This capability would be instrumental in accurately predicting changes in or recovery of FC, ultimately facilitating biomarker discovery and the development of individualized treatment strategies.

### 3.4 Limitations and future directions

#### 3.4.1 Linearity assumption and omitted higher-order effects

Despite its strengths, our generative model has several limitations. First, the assumption of linearity in SC-FC relationships, while computationally tractable, may oversimplify the nonlinear relationships, for example, between the fiber density and functional correlation, that underlie large-scale neural communication. Second, while our model incorporates indirect monosynaptic SC influences, it does not account for higher-order SC (e.g., Goñi et al., 2014). Future work should compare our framework forecast power against models with higher-order factors to establish the importance of higher-order SC connections.

#### 3.4.2 Challenges in validating personalized SC-FC rules

The observed lower accuracy in predicted FC using the group model likely reflects intersubject variability in the identified rules. This discrepancy may also arise from differences between group-level and subject-level estimation methods. However, it's important to consider that this variation could indicate subject-level overfitting despite our regularization efforts. This potential overfitting is a direct consequence of our framework's strength, which is providing accurate, individualized FC predictions through a data-driven generative approach. Therefore, to validate the subject-level rules, repeated measurements or causal studies, such as predicting post-surgical functional dynamics, are essential. It's important to acknowledge that the stability of SC-FC coupling rules over time or following surgical intervention is a confounding assumption for these approaches.

Subject-level estimation of *O* faces several interrelated limitations. First, the subject-level problem is underdetermined, with approximately 10,000 free parameters estimated from roughly 5,000 FC edge observations. Second, our λ-selection procedure, targeting a fixed parameter density via binary search, does not constitute cross-validated generalization in the classical sense. Third, the poor generalization seen with short FC windows in the BHA2 analysis reflects not only potential model overfitting but also the inherent instability of FC estimated from brief segments. Short windows are particularly sensitive to transient brain states and noise, and FC reliability increases substantially with scan length (Birn et al., 2013; Chang and Glover, 2010). Finally, proper out-of-sample validation would require repeated within-subject SC/FC acquisitions, which no currently available large-scale dataset provides. The primary protection against structural overfitting is the SC null model. Prediction accuracy drops dramatically when *O* is applied to edge-randomized or a different subject's SC, confirming that *O* encodes subject-specific structure-function coupling rather than arbitrary noise. Subject-level rules should be regarded as personalized FC fingerprints whose out-of-sample validity requires prospective validation with longitudinal datasets.

A further limitation concerns the linearity assumption. While computationally tractable and interpretable, the linear mapping *B* = *XOX*^*T*^ may miss non-linear SC-FC relationships, such as sigmoidal saturation in high-density tracts or threshold effects in weakly connected regions. Non-linear transfer functions applied to SC before entering the rule model could, in principle, reshape the identified cluster structure and the sign profile of the mediator class. Addressing this requires either a kernelized extension of the generative model or a systematic comparison against non-linear structural embeddings (e.g., sigmoid or tanh-transformed SC), which we identify as an explicit priority for future work.

The current analyses are based on a Schaefer 100-region cortical parcellation augmented with subcortical ROIs. Parcellation resolution is known to affect the mesoscale structure of both SC and FC matrices, and consequently the communities identified by WSBM. Finer parcellations introduce more regions in high-density cortical areas and may fragment or merge the identified clusters, potentially altering the specific regional composition of integrator and mediator classes. Coarser parcellations risk conflating structurally heterogeneous areas. Future work should evaluate the resolution-dependence of the structural taxonomy across multiple atlases (e.g., Schaefer 200, 400; Glasser HCP MMP1.0; AAL). We anticipate that the core block-sign grammar, specifically integrator clusters with positive inter-cluster weights and mediator clusters with negative weights, will be preserved across resolutions, while the precise anatomical composition of each cluster may vary.

The high subject-level accuracy (*R*^2^ = 0.81) is obtained when *O* is fitted and evaluated on FC estimated from the full resting-state time series. The BHA2 analysis uses short, non-overlapping segments to construct distinct training and test FC matrices. The accuracy decline at short windows reflects not only potential model-level overfitting but also the intrinsic unreliability of brief FC estimates. Short windows are dominated by transient brain states and physiological noise, and FC reliability is known to increase substantially with scan length (Birn et al., 2013). Moreover, resting-state FC is state-dependent, and shorter windows are more sensitive to spontaneous brain state fluctuations that may not be constrained by static SC (Chang and Glover, 2010; Allen et al., 2014). As window length increases, transient states average out and FC converges toward the SC-constrained steady state, which explains the progressive improvement in generalization seen in the test dataset.

#### 3.4.3 Toward modeling the dynamic support of FC by SC

In this work, we explored an alternative strategy for probing generalization to unseen subjects' FC using the BHA2 dataset, which provided around 15 minutes of resting-state time series. Our findings indicate that substantially longer time series are necessary to determine an FC estimation window size that promotes generalizability across unseen FC. Despite this, we observed that shorter windows, while generally showing poor generalizability, yielded highly accurate predicted FC in the training dataset.

This suggests that while static SC-FC mapping may be achievable, long-duration correlation-based FC might not fully capture the time-varying support of FC by SC. Instead, our results imply that SC may precisely explain how dynamic brain activity is supported and emerges from SC. This would necessitate a time-varying *O* matrix, illustrating how specific SC components actively contribute to ongoing FC, directly or indirectly. This opens a promising research direction for investigating dynamic rule matrices and their role in explaining time-varying brain activity.

#### 3.4.4 Incorporating regional microstructural and molecular factors

In this work, for the dataset without information on ROI self-connection fiber counts (i.e., BHA2), we assumed a uniform value for the structural self-coupling parameters across all brain regions. However, future work can improve the prediction of our model by informing these parameters by integrating regional information that affects functional activity. For example, local microstructural integrity metrics measured with advanced diffusion MRI metrics such as Neurite Orientation Dispersion and Density Imaging (Kraguljac et al., 2023), or molecular influences through genetic (Hawrylycz et al., 2012) and neurotransmitter profiling (Hansen et al., 2022).

#### 3.4.5 Extending to directed and causal connectivity measures

Finally, we examined correlation-based FC and demonstrated that SC can shed light on the origins and signs of certain spurious correlations. Future research should build upon this framework by incorporating effective connectivity measures, such as linear time-invariant models (Ashourvan et al., 2022) and dynamic causal modeling (Friston et al., 2003). These methods provide valuable insights into the directed and causal influences between brain regions and deepen our understanding of how the structural connectome drives functional dynamics.

### 3.5 Conclusion

Our study introduces a novel generative framework that bridges statistical and mechanistic modeling approaches by formalizing structure-function coupling as an explicit mathematical relationship (*B* = *XOX*^*T*^). This framework advances beyond existing methods by decomposing functional connectivity into interpretable structural rules that capture direct anatomical connections and monosynaptic motifs, while achieving high predictive accuracy through subject-specific parameterization.

The identification of brain regions as structural *linchpins* and *fulcrums* provides a new organizational taxonomy for understanding how local anatomical features scale to global functional dynamics. Our findings reveal that functional connectivity emerges primarily from direct structural connections rather than abstract global topological properties, challenging prevailing assumptions about polysynaptic communication.

Clinically, our framework offers a principled approach for predicting functional reorganization following structural disruptions. The demonstration that pathological changes involve reorganization of coupling *rules* themselves – not just structural alterations – opens new avenues for precision medicine applications in neurology and psychiatry. By providing a mechanistic understanding of how specific structural disruptions give rise to particular functional deficits, this work lays the groundwork for the next generation of structure-informed interventions in brain health and disease.

## 4 Materials and methods

### 4.1 Data and preprocessing

We utilized publicly available datasets, providing subject-level structural and functional connectivity matrices derived from diffusion imaging and resting-state functional MRI.

The dataset that we analyzed in the main manuscript includes 50 healthy adults (23 female; mean age 29.5 ± 5.6 years; 47 right-handed) (Royer et al., 2022). SC matrices were generated via anatomically constrained tractography and SIFT2-weighted streamlines (Smith et al., 2015), and FC matrices were computed by Pearson correlation of parcellated rs-fMRI time series. More details on the imaging protocol and data preprocessing can be found in Royer et al. (2022).

We also leveraged a second dataset that consists of 27 healthy adults (35 ± 6.8 years) and 27 schizophrenia-spectrum patients (41 ± 9.6 years), all scanned at 3 T (Siemens Trio) and anonymized with ethics approval from the University of Lausanne (Griffa et al., 2017; Daducci et al., 2012). Precomputed SC and FC matrices were obtained from diffusion spectrum imaging and eyes-open resting-state fMRI, respectively, using the Connectome Mapper pipeline. The SC matrix elements represent the number of streamlines running between the regions, adjusted for average streamline length and regional sizes, and FC matrices were computed by Pearson correlation of parcellated rs-fMRI time series.

Finally, we used a third dataset—Brain Hierarchical Atlas 2 (BHA2) (Jimenez-Marin et al., 2024) – that, in addition to SC and FC, provided subjects' resting-state time series, which we used to explore the effect of model parameters' sparsity on FC prediction accuracy (for more details, see see Section 4.3). This dataset includes 136 healthy young adults (aged 20-30) from the MPI-LEMON cohort (Jimenez-Marin et al., 2024; Babayan et al., 2019). We used the provided SC and FC matrices constructed from preprocessed DWI and rs-fMRI data, using a 183-region parcellation from hierarchical clustering. Each entry in the SC matrices represents the number of stream counts between two regions, and each entry in the FC matrices represents the functional correlation between two regions.

### 4.2 Group-level model solution

For the group-level fits, for each subject *i*∈{1, 2, …, *n*}, we have the equation ***B***_*i*_ = ***X***_*i*_***OX***_*i*_, where $B_{i},X_{i},O\in {\mathbb{R}}^{N\times N}$ represent the FC matrix, SC matrix, and rule matrix, respectively, with *N* denoting the number of brain regions. Thus, the optimal rules matrix, at the group level, for the set of individuals is the matrix, ***O***, that minimizes

$$\|\sum_{i}\left(X_{i}OX_{i}-B_{i}\right){\|}_{2}.$$

However, by definition, the rules matrix ***O*** must be symmetric. To ensure that the solution to this minimization problem is symmetric, we define ***K*** = ***X***⊗***X*** and modify ***K*** and ***O*** using the following algorithm – for a visual explanation, see Supplementary Figure 3:

1. Let *L* = {(*i, j*)∈ℕ × ℕ∣*i*<*j*<*n*}, where *n* denotes the number of rows (columns) of ***X***;
2. Index ***K*** starting at 0;
3. For each (*i, j*)∈*L*, add the (*in*+*j*)th column of ***K*** to the (*jn*+*i*)th column of ***K***;
4. For all (*i, j*)∈*L*, delete the (*in*+*j*)th column of ***K***the corresponding element of vec(***O***) simultaneously.

Finally, denoting the transformed matrices as $\tilde{K}$ and $\text{vec}\left(\tilde{O}\right)$, we can write

$$\begin{array}{l} \text{vec}\left(\tilde{O}\right)={\left(\begin{array}{c} {\tilde{K}}_{1}\\ {\tilde{K}}_{2}\\ {\tilde{K}}_{3}\\ \vdots \\ {\tilde{K}}_{n} \end{array}\right)}^{†}\left(\begin{array}{c} \text{vec}\left(B_{1}\right)\\ \text{vec}\left(B_{2}\right)\\ \text{vec}\left(B_{3}\right)\\ \vdots \\ \text{vec}\left(B_{n}\right) \end{array}\right), \end{array}$$

where † represents the Moore-Penrose pseudoinverse. No regularization is needed because the matrix formed by ${\tilde{K}}_{1},...,{\tilde{K}}_{n}$ was consistently found to be full rank. The matrix ***O*** can then be easily recovered, as $\tilde{O}$ simply contains the upper triangular elements of ***O***.

### 4.3 Subject-level fitting

For each subject-level fit, the equation ***B***_*i*_ = ***X***_*i*_***O***_*i*_***X***_*i*_ is underdetermined. To account for this, we utilize LASSO regression (Tibshirani, 1996). The regularization parameter λ∈ℝ^+^ controls the sparsity of the solution. The objective function, for each subject *i*, now becomes

$$\begin{array}{l} \frac{1}{2}||\left(X_{i}OX_{i}-B_{i}\right)|{|}_{2}+\lambda ||O_{i}|{|}_{1}. \end{array}$$

After undergoing the same transformation used for the group-level fits, described in Section 4.2, we can now rewrite the problem as $vec\left(B_{i}\right)={\tilde{K}}_{i}vec\left(O_{i}\right)+\lambda |vec\left(O_{i}\right)|{|}_{1}$ (see Supplementary Figure 8).

#### 4.3.1 Challenges in cross-validation for global models

The regularization term, λ, dictates the sparsity of model parameters; a higher λ value results in models with fewer active parameters. This promotes generalization and helps prevent overfitting, particularly in models with a large number of parameters. Ideally, λ is selected via cross-validation on an independent test dataset.

However, our model uniquely leverages the relationships between all variables to predict FC. This characteristic prevents the standard practice of splitting data into training and testing subsets, as it would disrupt the global variable relationships essential for our model's operation. Thus, traditional cross-validation would require datasets with repeated SC and FC measures, which were unavailable for this study.

#### 4.3.2 Alternative generalizability assessment using BOLD time series

Given the limitations of traditional cross-validation and recognizing the arbitrary nature and potential inconsistency of criteria like AIC and BIC, we pursued an alternative approach to evaluate model generalizability. We posited that repeated DWI scans, acquired within a short temporal window (hours), would not reflect actual changes in SC. Therefore, our focus was to assess the model's generalization to unseen FC. As the Royer et al. (2022) dataset did not contain repeated FC measures, we utilized the BHA2 dataset. This dataset provided BOLD time series for ROIs, enabling the construction of distinct training and test FC matrices. Hence, it allowed us to investigate the impact of the FC estimation window length on model performance.

#### 4.3.3 Regularization parameter effects on model performance

We evaluated the effect of the λ parameter on model performance using three sample subjects, presenting results for the training and test FC datasets in Supplementary Figures 9a, b, respectively. Consistent with expectations, higher λ values led to sparser rule matrices and diminished model performance.

In the training dataset (Supplementary Figure 9a), models generally performed best with larger FC estimation windows, though short windows yielded high accuracy for two subjects. Performance consistently dropped when rule matrix density fell below 60-80%. The test dataset (Supplementary Figure 9b) showed similar patterns, but with a more pronounced performance reduction for smaller window sizes.

#### 4.3.4 Binary search strategy for regularization parameter selection

For regularization to clearly enhance generalization, peak performance should occur at lower densities (i.e., where regularization has pruned parameters). While some peaks appeared at densities below full for window sizes under 274 seconds, the overall model performance in these cases was already poor. Thus, despite observing limited signs of generalizability, the evidence was insufficient to guide the selection of the regularization parameter in this study.

Consequently, the λ was determined through a binary search, targeting a density of 0.8 ± 0.01 non-zero elements within the *O* matrix. This specific density was chosen as it yielded acceptable performance on the training data and offered a degree of generalizability to unseen FC. This binary search was initialized with a high and low value set to $\lambda_{max}=\frac{1}{N}|\langle x_{i}^{k},vec\left(B_{i}\right)\rangle$ and $\lambda_{min}=\frac{\lambda_{max}}{100000}$. This maximum lambda value represents the value at which the fitted ***O*** matrix becomes all zeroes (Friedman et al., 2010). A 0.01 tolerance for the density was used so that binary search could converge to a λ value in a reasonable number of steps.

### 4.4 Model performance evaluation

To evaluate the model's ability to predict empirical FC, we performed a linear regression between the predicted and observed FC values. Specifically, we regressed the upper triangular elements of the predicted FC matrix against those of the actual FC matrix for each subject. An intercept term was included to account for potential offsets in predicted FC values. Model performance was quantified using the coefficient of determination (*R*^2^), reflecting the proportion of variance in observed FC explained by the model predictions.

### 4.5 Statistical analysis and null models

For each subject's FC matrix, 100 null matrices were generated, preserving the original degree and strength distributions. Randomization followed the method introduced by Rubinov and Sporns (2011), using the Brain Connectivity Toolbox null_model_und_signed function, with the weight frequency parameter set to 1 and the bin swaps parameter set to 10. Next, for each subject *i*, 100 null rule matrices were computed, one for each null FC matrix, using lasso regression with the same penalty term, λ, that was selected in training the given subject. Finally, at the group level, 100 null rule sets were computed as follows:

$$\begin{array}{l} vec\left(O_{i}\right)={\left(\begin{array}{c} {\tilde{K}}_{1}\\ {\tilde{K}}_{2}\\ {\tilde{K}}_{3}\\ ...\\ {\tilde{K}}_{n}\; \end{array}\right)}^{†}\left(\begin{array}{c} vec\left(B_{i1}\right)\\ vec\left(B_{i2}\right)\\ vec\left(B_{i3}\right)\\ ...\\ vec\left(B_{in}\right)\; \end{array}\right), \end{array}$$

where each ${\tilde{K}}_{i}$ is computed in the same manner as in Section 4.2, but where each ***B***_*ij*_ is instead the *j*th null FC matrix for subject *i*.

These null rules were then utilized to determine statistically significant rules at both the subject and group levels. A Wilcoxon signed rank test was conducted to compare the (*i, j*)th element of the rules matrix with the corresponding elements in each of the 100 null rules matrices. FDR correction was then applied to the resultant *p*-values to correct for multiple comparisons. Finally, rules whose associated *p*-values were less than 0.05 were kept. The remaining rules were set to 0.

### 4.6 Virtual resections

For each region of interest, all structural connections not involving the region or system of interest were removed. For a given region or system of interest, constructed of some subset of the parcellated regions, all elements of ***X*** except for those of the form ***X***_*ij*_ and ***X***_*ji*_ were set to 0, where *i* belongs to the set of indices belonging to the region or system of interest and *j* belongs to the set of all indices. This procedure was performed on the matrix ***X*** for each subject for each region of interest. We then calculated the element-wise mean of each subject's predicted FC under the subject's modified ***X*** matrix. Two sets of these predictions were made both under the subject and group-level rules.

### 4.7 Search information measure

Search information is a network-theoretic metric that quantifies how difficult it is to navigate between two nodes in a graph when only local information is available at each step. Originally introduced in Goñi et al. (2014), it has been widely used in brain network analysis to evaluate communication efficiency along shortest paths between regions of interest.

Given a weighted, undirected network (e.g., a structural connectivity matrix), the search information *S*_*ij*_ between nodes *i* and *j* is defined as the negative logarithm of the probability of successfully following a shortest path from *i* to *j* using only local knowledge of each node's neighbors, i.e.,

$$\begin{array}{l} S_{ij}=-\log_{2}P_{ij}, \end{array}$$

where *P*_*ij*_ is the product of transition probabilities along the shortest path π(*i*→*j*). At each node *k* along this path, the probability of selecting the correct next step *k*→*k*′ is computed by normalizing the edge weights among *k*'s neighbors:

$$P_{ij}=\prod_{\left(k\to k^{\prime })\in \pi (i\to j\right)}\frac{w_{kk^{\prime }}}{\sum_{m\in N(k)}w_{km}}.$$

Here, $w_{kk^{\prime }}$ is the weight of the edge between nodes *k* and *k*′, and N(k) denotes the set of neighbors of node *k*. This formulation assumes that at each step, the traveler has no global map and chooses the correct neighbor based only on edge weights.

Search information thus reflects the *navigational cost* of traversing the shortest path under local constraints. A low value indicates that the path is easily discoverable with local knowledge, while high values suggest that accurate navigation requires more information (Goñi et al., 2014).

### 4.8 Weighted stochastic block model

We used a weighted stochastic block model (WSBM) to identify communities in our weighted, sparse rule matrix. Let the network be represented by its adjacency matrix, ***O***∈ℝ^*N*×*N*^, where ***O***_*ij*_ denotes the weight of the rule from node *j* to node *i*. We assume that each node *i* belongs to one of *K* distinct communities, denoted by the label *z*_*i*_∈{1, …, *K*}.

In the traditional (binary) stochastic block model, an edge between nodes *i* and *j* exists with probability θ_*z*_*i*_*z*_*j*__, where θ_*rs*_ is the probability of connection between communities *r* and *s*. Formally, for an unweighted (binary) network, the likelihood of observing ***O*** under this model is given by

$$\begin{array}{l} P\left(O\left\{\theta_{rs}\right\},\left\{z_{i}\right\}\right)=\underset{i,j>i}{\prod }\theta_{z_{i}z_{j}}^{O_{ij}}{\left(1-\theta_{z_{i}z_{j}}\right)}^{1-O_{ij}}, \end{array}$$

where ***O***_*ij*_∈{0, 1}. By maximizing this likelihood with respect to the community labels {*z*_*i*_} and probabilities {θ_*rs*_}, one can uncover an arrangement of nodes into communities that capture their underlying connection patterns.

Many real-world networks, including our rule matrix, are weighted and potentially sparse. To accommodate real-valued edge weights, one can adopt an exponential family version of the block model. In general, the likelihood function can be expressed as

$$\begin{array}{l} P\left(O\left\{\theta_{rs}\right\},\left\{z_{i}\right\}\right)\propto \exp\left(\underset{i,j}{\sum }T\left(O_{ij}\right)\cdot \eta \left(\theta_{z_{i}z_{j}}\right)\right), \end{array}$$

where *T*(·) are sufficient statistics for the chosen distribution (e.g., a Gaussian, log-normal, etc.), and η(·) are their corresponding natural parameters. The parameters θ_*z*_*i*_*z*_*j*__ thus characterize how any two communities, *z*_*i*_ and *z*_*j*_, tend to connect in terms of both the existence and the magnitudes of edges.

In the WSBM, as in the classical SBM, each node's community assignment {*z*_*i*_} and the parameters θ_*z*_*i*_*z*_*j*__ fully specify the model. The key difference is that θ_*z*_*i*_*z*_*j*__ now describes the distribution of edge weights rather than just their presence or absence. Following the approach in (Aicher et al. 2013) and (Betzel et al. 2018), we assume these weights are drawn from a normal distribution with sufficient statistics *T* = (*x, x*^2^, 1) and natural parameters $\eta =\left(\frac{\eta }{\sigma^{2}},-\frac{1}{2\sigma^{2}},-\frac{\mu^{2}}{2\sigma^{2}}\right)$. Thus, an edge between communities *z*_*i*_ and *z*_*j*_ is characterized by a mean μ_*z*_*i*_*z*_*j*__ and variance $\sigma_{z_{i}z_{j}}^{2}$. The likelihood can then be written as

$$P\left(A\left\{z_{i}\right\},\left\{\mu_{rs}\right\},\left\{\sigma_{rs}^{2}\right\}\right)=\underset{i,j}{\prod }\exp\left(A_{ij}\frac{\mu_{z_{i}z_{j}}}{\sigma_{z_{i}z_{j}}^{2}}-\frac{A_{ij}^{2}}{2\sigma_{z_{i}z_{j}}^{2}}-\frac{\mu_{z_{i}z_{j}}^{2}}{2\sigma_{z_{i}z_{j}}^{2}}\right).$$

We maximize the likelihood of this sparse WSBM using a Variational Bayes technique described in Aicher et al. (2013) and implemented in MATLAB using code (WSBM V1.2) made available on the author's personal website (https://aaronclauset.github.io/wsbm/). This inference procedure jointly estimates the parameters {θ_*rs*_} for each pair of communities and the assignment {*z*_*i*_} of each node, thereby offering a flexible framework for detecting multiple forms of mesoscale organization in weighted networks.

Due to the non-deterministic nature of the algorithm, we performed 10 independent runs with randomized initializations and selected partitions, maximizing the log evidence. To enhance interpretability, we selected a partition (*k* = 4) for the manuscript figure that visually emphasized intuitive mesoscale rules (e.g., clear core-periphery distinctions). While our analysis focused on four clusters, future work should systematically characterize subject-specific rule matrices across different clustering resolutions. Such an exploration could deepen understanding of the mesoscale diversity of structural rules.

#### 4.8.1 Subject-level WSBM and inter-individual consistency

For the inter-individual reliability analysis (Section 2.2.3), WSBM was applied independently to each subject's *O* matrix using the same settings (*k* = 4, normal weight distribution, α = 0, 10 independent trials, best log-evidence solution retained). Subject-level community labels were aligned to the group partition via the Hungarian algorithm (Kuhn, 1955), which minimizes the cost of a bipartite assignment between group and subject label sets. Partition similarity was quantified by normalized mutual information (NMI) (Strehl and Ghosh, 2002), computed for every pair of aligned subject partitions. A null distribution of NMI values was constructed from 100 permutations of the group partition labels. A one-tailed *t*-test confirmed that observed pairwise NMI significantly exceeded the null mean. Block-sign consistency was assessed by computing the mean weight of each *k*×*k* inter-community block across subjects and testing it against zero with a one-sample *t*-test; *p*-values were FDR-corrected (Benjamini–Hochberg) across all block pairs. Node-level assignment consistency was defined as the fraction of subjects assigning each node to the same cluster as the group solution; differences across clusters were tested with the Kruskal–Wallis non-parametric one-way ANOVA.

## Data Availability

Publicly available datasets were analyzed in this study. This data can be found here: https://www.nature.com/articles/s41597-022-01682-y, https://zenodo.org/records/3758534, and https://zenodo.org/records/8158914.
