# Supplementary material for: The structural grammar of integration and competition in the human connectome
Source: Front Comput Neurosci. 2026 Jun 24;20:1810942. doi: 10.3389/fncom.2026.1810942 (PMC13341858; doi:10.3389/fncom.2026.1810942)
Supplement: Supplementary file 1 [file Supplementary_file_1.pdf]

frontiers

Supplementary Material for ”The Structural Grammar of Integration and Competition in the Human Connectome”

1 SUPPLEMENTARY FIGURES

1.1 Figures

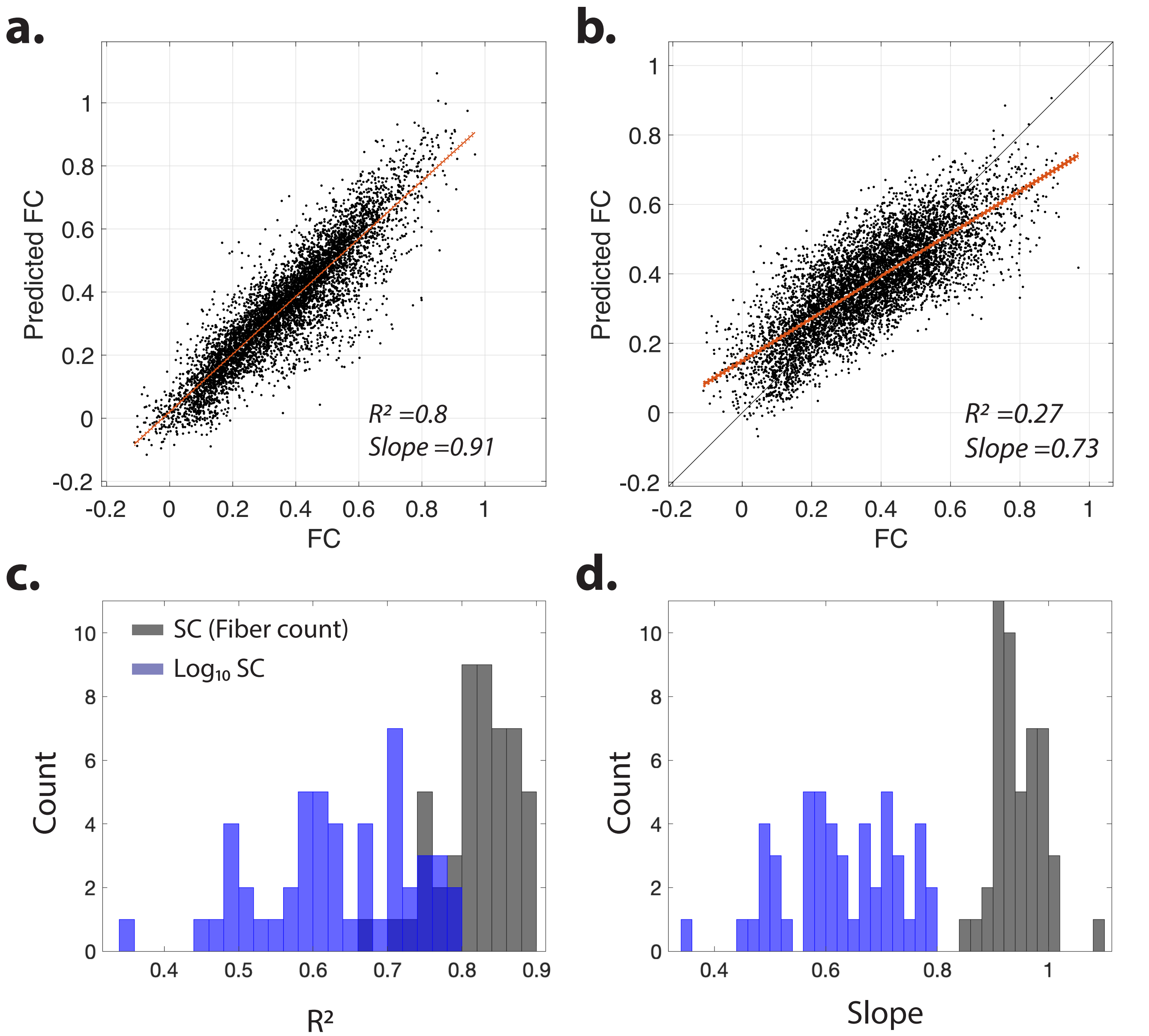

Figure S1. Effect of Log-transforming SC on the prediction of FC (a) Scatter plot comparing the sample subject's actual FC values with the predicted FC values using the subject-specific rule matrix derived from the SC matrix of fiber counts between brain regions. The red line represents the linear fit, while the dashed line indicates the 95% confidence interval. **(b)** Same as panel **(a)**, except we used $\log_{10}(\text{SC})$. To avoid issues with zero values, we added 1 to all elements of the SC matrix before applying the logarithm. **(c)** Distribution of R^2 values across all subjects ($n = 50$) for the linear regression between actual and predicted FC values using subject-specific rule matrices derived from fiber count SC (black) and $\log_{10}(\text{SC})$ (blue). Predictions using $\log_{10}(\text{SC})$ result in a significant reduction in the mean goodness-of-fit (R^2) of the linear regression (t-test, $p = 4.8 \times 10^{-18}$). **(d)** Distribution of slope values across all subjects ($n = 50$) for the linear regression between actual and predicted FC values using subject-specific rule matrices derived from fiber count SC (black) and $\log_{10}(\text{SC})$ (blue). Predictions using $\log_{10}(\text{SC})$ lead to a significant reduction in the mean slope of the linear regression (t-test, $p = 2 \times 10^{-38}$).

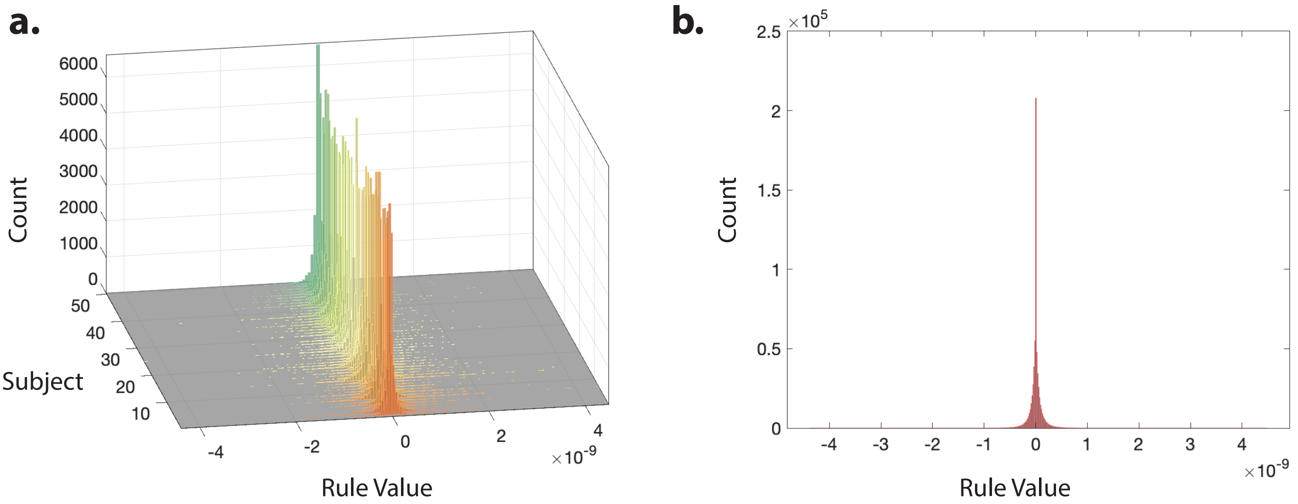

Figure S2. Distribution of rule values(a) Distributions of subject-level rule matrix element values, color-coded for each subject. **(b)** Distribution of subject-level rule matrix element values aggregated across all subjects.

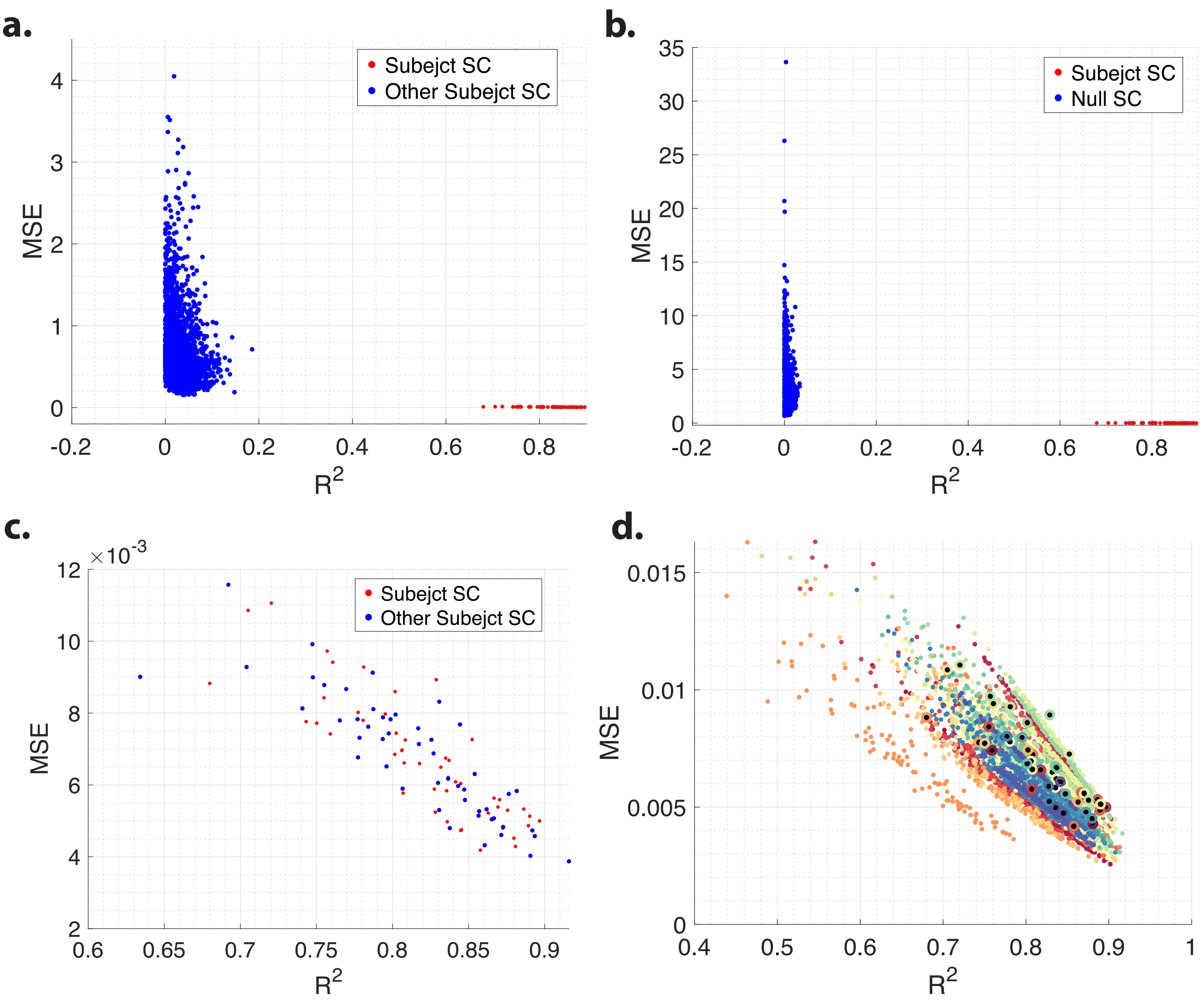

Figure S3. FC prediction using other subjects and null SC (a) Goodness-of-fit for the linear regression between predicted and actual FC, evaluated using mean squared error (MSE) and R^2 values. Predictions based on each subject's SC are shown in red, while those using another subject's SC and the original subject's estimated rule matrix O are shown in blue. **(b)** Same as panel **a**, except the blue dots represent predictions generated from SC randomized null models ($n = 100$ nulls per subject) **(c)** Same setup as panel **a**, but here the rule matrix was re-estimated using a randomly selected subject's SC to predict the original subject's FC (blue dots). **(d)** Similar to panel **b**, except rule matrices were re-estimated for each SC randomized null model as in panel **c**. Subjects are color-coded, and actual predictions based on each subject's own SC and FC are indicated with larger markers featuring a black center.

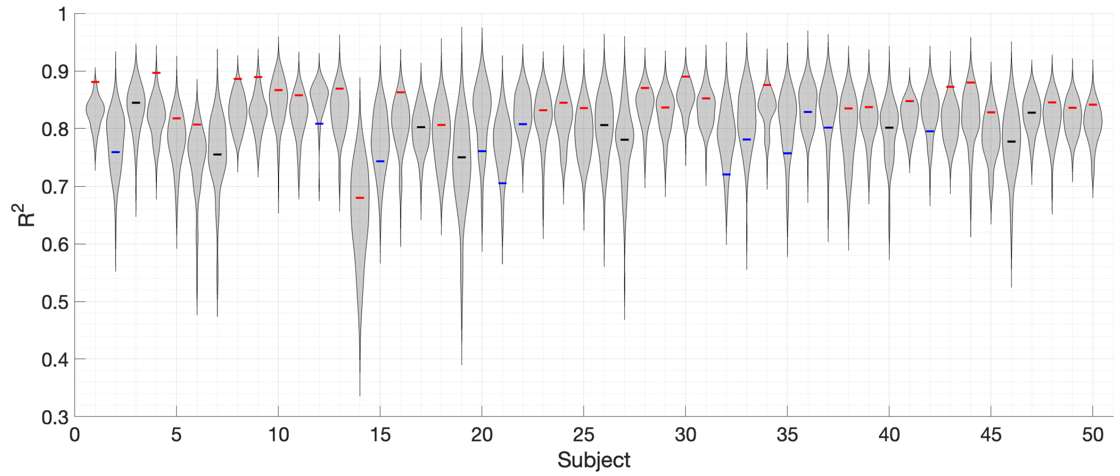

Figure S4. FC prediction using null SC (a) Goodness-of-fit for the linear regression of actual versus predicted FC, assessed using R^2 values. R^2 values for the Predictions based on each subject's own SC are shown using the bar, and the violin plots show the R^2 values for predictions based on SC randomized null models, with rule matrices estimated from these SC nulls. The significantly ($t-$test,$p < 0.05$, FDR corrected for multiple comparisons across subjects) higher or lower R^2 values than predicted based on SC randomized null models are color-colored in red and blue, respectively.

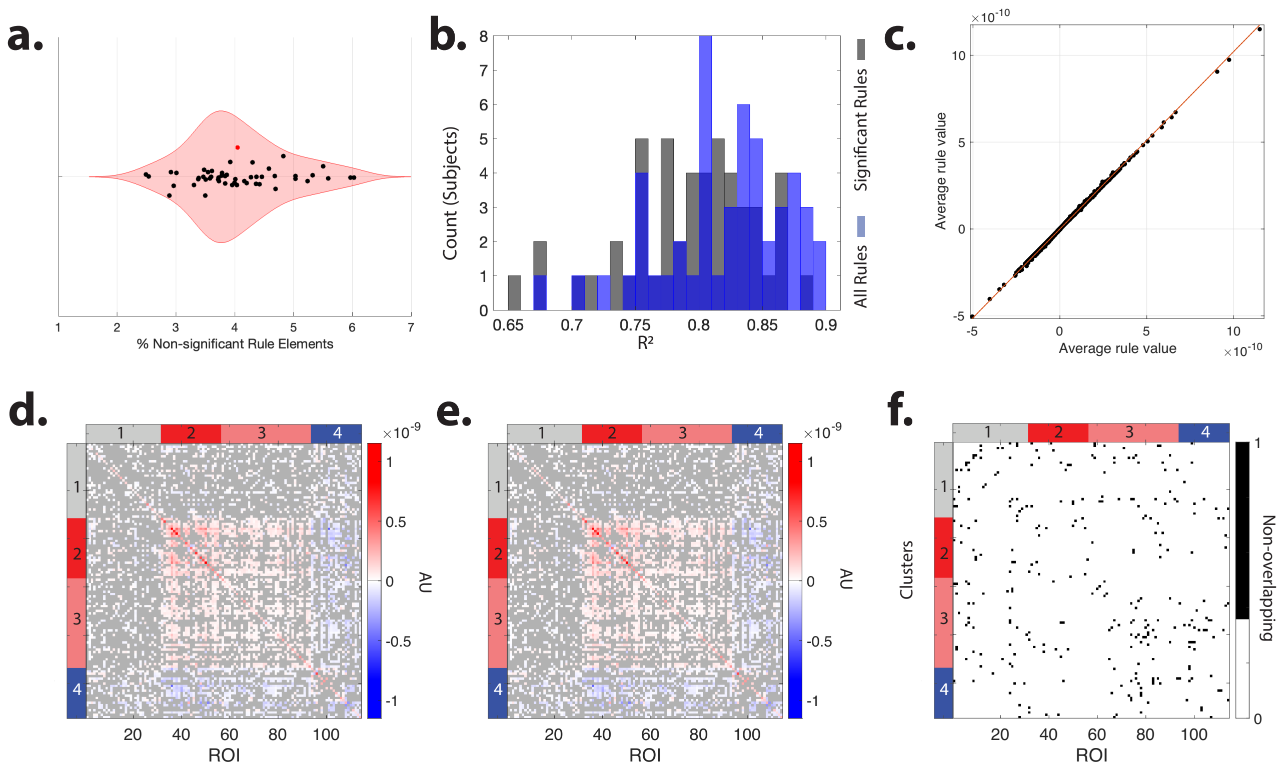

Figure S5. FC prediction using subject's unique rules (a) The percentage of non-significant rules (nonparametric permutation testing using randomized FC nulls, $p < 0.05$, FDR-corrected for multiple comparisons – see Materials and Methods section for details) relative to the total number of rules for each subject. The red dot shows the average percentage across all subjects. **(b)** Distributions of the R^2 values of the linear fit between the actual and predicted FC values using all subject-level rules (black) and using only the significant rule matrix elements (blue). Removing non-significant rules that are not significant for each subject results in a significant ($t-$test, $p = 1.01 \times 10^-14$) reduction in the mean goodness-of-fit of a linear fit (R^2), comparing the actual versus predicted FC values. **(c)** The group-average subject-level rule values against the rule values after removing each subject's non-significant rules. Red line shows the linear fit (slope $= 1.02$, $R^2 = 0.99$) **(d)** Subject-level rule matrix O averaged across all subjects, with brain regions sorted by their cluster assignments identified using the WSBM method ($k = 4$). The rule elements with means that show no significant ($t-$test, $p < 0.05$, FDR corrected for multiple comparisons across all rules) difference from zero across all subjects are color-coded in gray. **(e)** Same matrix in panel **d**, except the non-significant subject-level rules were removed before the calculation of group means. **(f)** The overlap between the matrices in panels **c** and **d** marked by white and black.

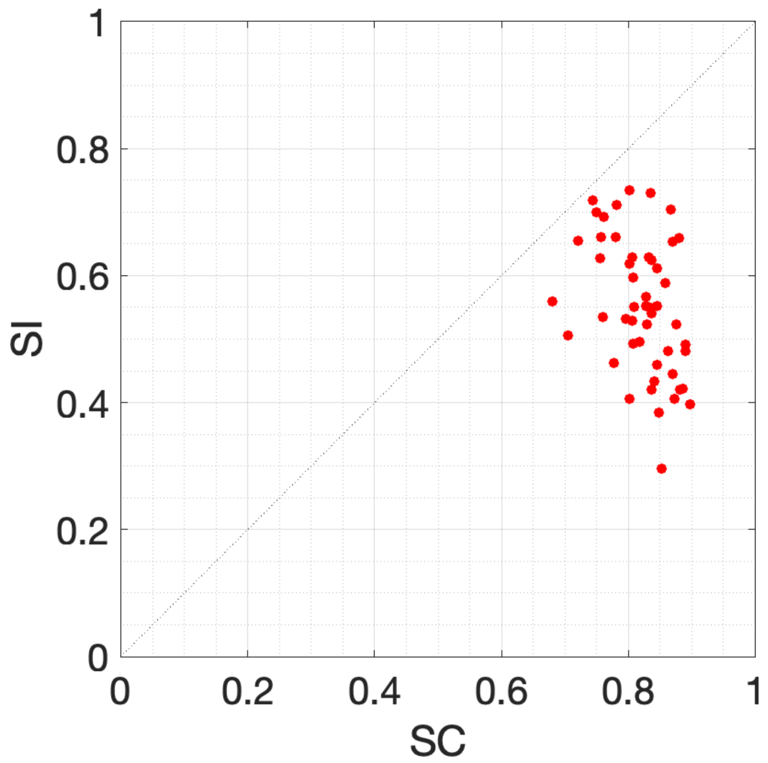

Figure S6. Search Information versus structural connectome prediction accuracy. The plot represents the goodness-of-fit for the linear regression of actual versus predicted FC, assessed using R^2 values, for the Predictions based on each subject's own SC compared to those using the search information matrix derived from the subject's SC. The R^2 values are significantly higher when subjects' SC was used compared to the search information matrix to predict FC matrices ($t-$test, $p = 8.65 \times 10^-19$).

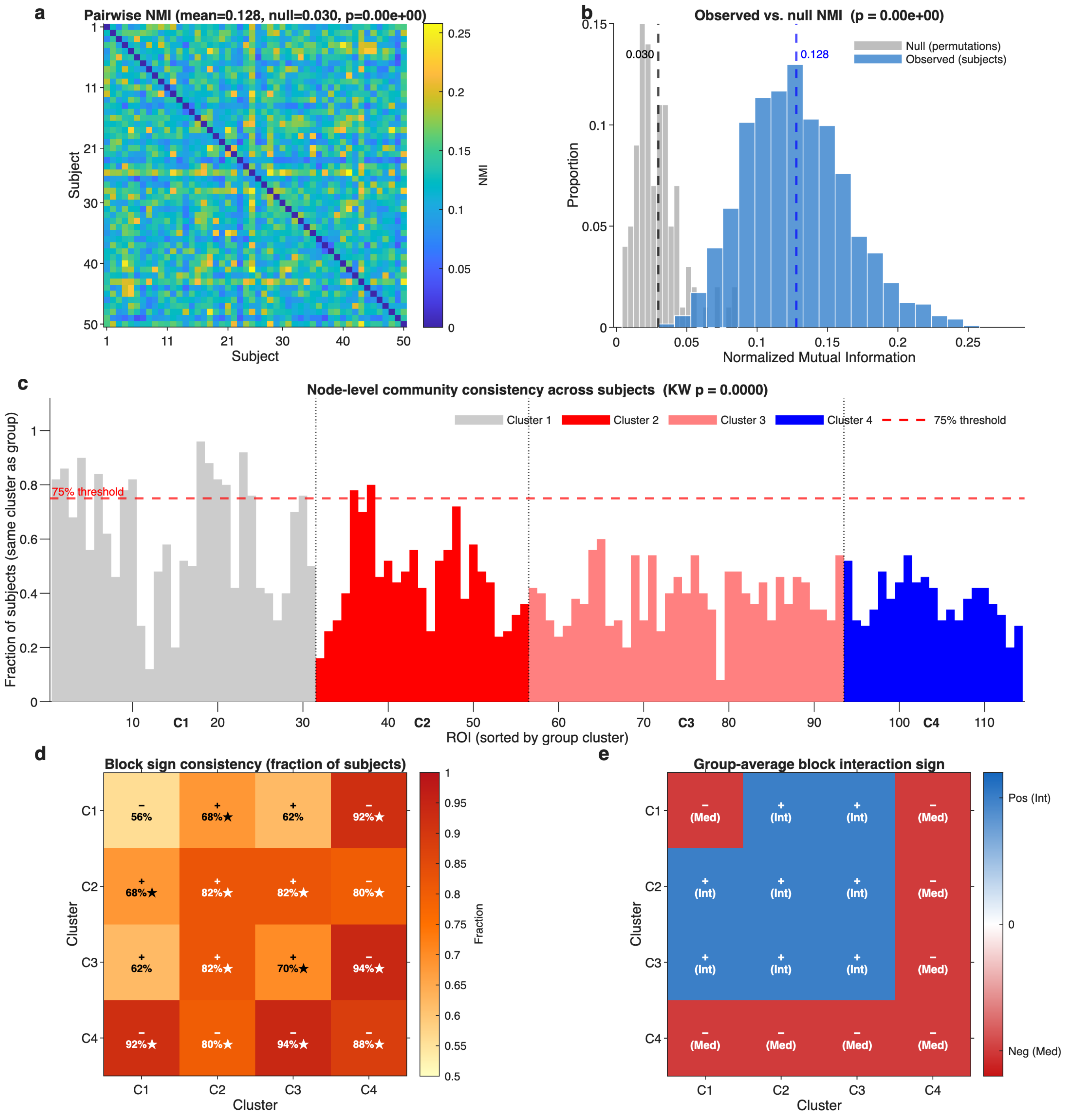

Figure S7. Subject-level WSBM analysis. (a) Pairwise normalized mutual information (NMI) matrix across all 50 subjects (mean = 0.128, null = 0.030, $p < 10^{-30}$). **(b)** Distribution of observed pairwise NMI (blue) versus null NMI from permuted labels (grey); dashed lines mark distribution means. **(c)** Node-level community consistency: fraction of subjects assigning each ROI (sorted by group cluster) to the same cluster as the group solution; red dashed line marks the 75% threshold; Kruskal-Wallis $p < 0.0001$ across clusters. **(d)** Block-sign consistency heatmap (fraction of subjects with the same signed block mean as the group); stars (★) mark FDR-significant blocks (t-test, FDR $q < 0.05$); annotations show sign and percentage. **(e)** Group-average block interaction sign (+ = integrator, − = mediator).

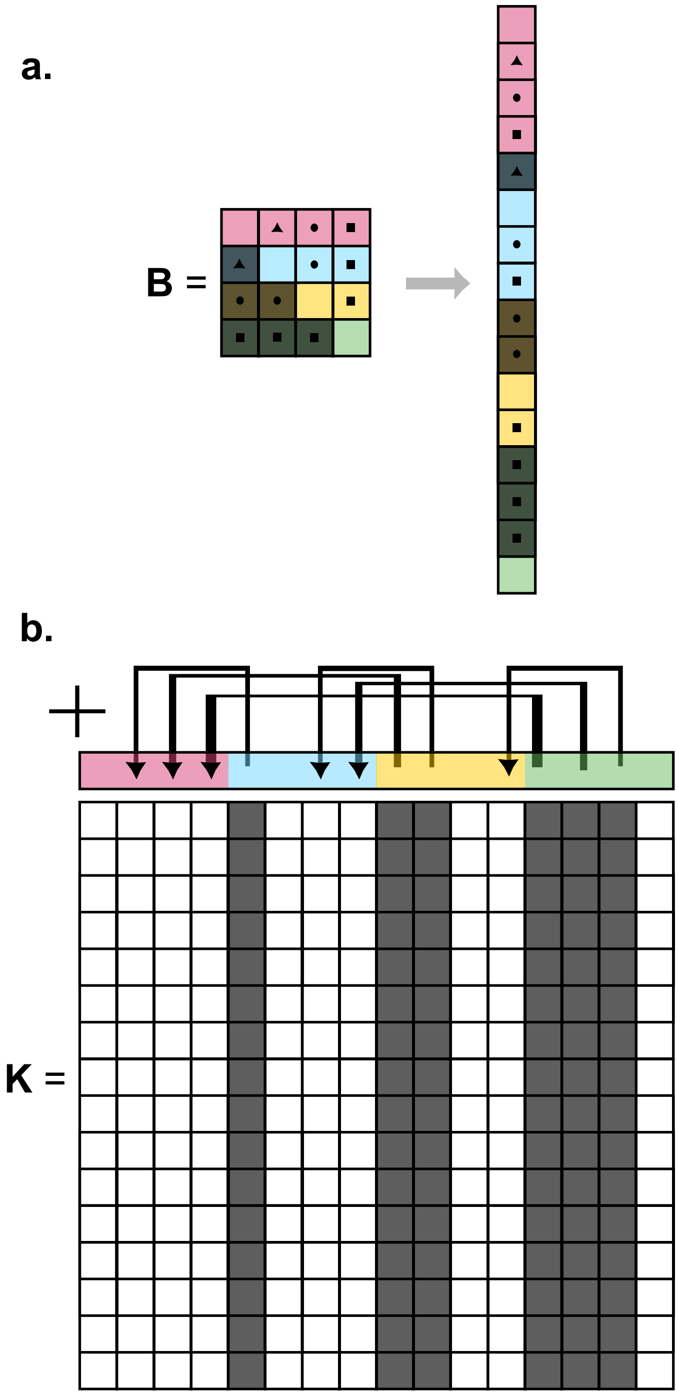

Figure S8. schematic of algorithm to preserve symmetry The above figure is a visual representation of the algorithm in section 4.2 used to modify $\boldsymbol{K}$ and $\boldsymbol{B}$. **(a)** $\boldsymbol{B}$ undergoes the transformation $vec(\boldsymbol{O})$, then the grayed values are removed. **(b)** Each grayed column of $\boldsymbol{K}$ is added to the column to which its arrow points. The grayed columns are then deleted from $\boldsymbol{K}$.

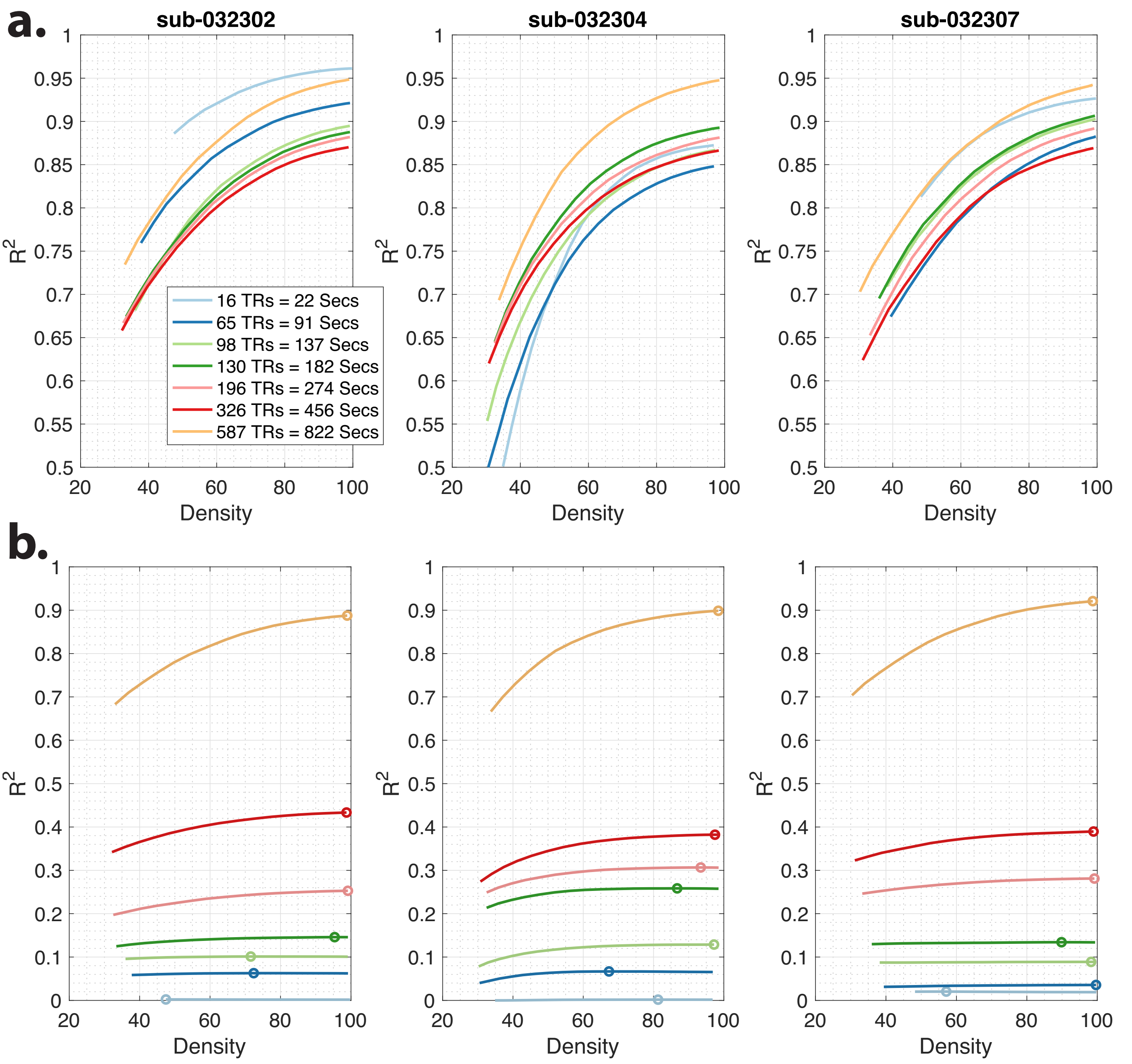

Figure S9. The effect of rule matrix density on prediction accuracy. (a) Prediction accuracy (R^2) of the linear-regression model versus rule-matrix sparsity (percentage of nonzero entries), shown for color-coded window sizes corresponding to approximately 2 %, 10 %, 15 %, 20 %, 30 %, 50 % and 90 % of the time series length. **(b)** Same as panel **a**, but prediction accuracy is evaluated against FC matrices computed from unseen (test) time series. Maximum values are marked by 'o'. Note that the 90 % window size overlaps with the training time series and is the only condition not evaluated on fully unseen data.
